# Accurate Quantification of Multifunctional C_2–3_ Organosulfates in Atmospheric Aerosols Using Liquid Chromatography-Electrospray Ionization Mass Spectrometry: Overcoming Matrix Effects and Underestimation

**DOI:** 10.1021/acs.est.5c01846

**Published:** 2025-06-17

**Authors:** Shumin Liang, Yuchen Wang, Hanzhe Chen, Wan Chan, Jian Zhen Yu

**Affiliations:** † Department of Chemistry, The Hong Kong University of Science and Technology, Kowloon 999077, China; ‡ College of Environmental Science and Engineering, Hunan University, Changsha, Hunan 410082, China; § Division of Environment and Sustainability, The Hong Kong University of Science and Technology, Kowloon 999077, China

**Keywords:** secondary organic aerosol, C_2−3_ organosulfates, quantitative analysis, reversed-phase liquid chromatography, hydrophilic interaction liquid chromatography, matrix effects

## Abstract

Reversed-phase liquid chromatography (RPLC) coupled with electrospray ionization-mass spectrometry (ESI-MS) is widely used to analyze polar organic compounds in atmospheric particulate matter (PM). However, its efficacy for small, polar multifunctional C_2_–C_3_ organosulfates (C_2–3_OSs)—conceivably key products of isoprene oxidation—is questionable. Notable matrix effects are anticipated to arise from poor retention and coelution with abundant salts in PM samples. Here, we systematically evaluated RPLC versus hydrophilic interaction liquid chromatography (HILIC) coupled with ESI-Orbitrap MS in quantifying PM-bound C_2–3_OSs. We synthesized three C_2–3_OSs, including glycolic acid sulfate, hydroxyacetone sulfate, and lactic acid sulfate. The availability of authentic standards enabled the first quantitative assessment of measurement bias for C_2–3_OSs using the RPLC-ESI-Orbitrap MS method, revealing an underestimation of these compounds by 1–2 orders of magnitude. The measurement bias primarily stemmed from the matrix effects arising from the coexisting bisulfate in ambient PM. In contrast, HILIC notably outperformed RPLC in retentive capacities and peak resolving abilities, effectively avoiding matrix suppression effects. Additionally, the HILIC-ESI-MS method uncovered six previously unreported C_2–3_OSs, expanding our knowledge of atmospheric OSs. This work enhances our capability of accurate quantification of aerosol components, thus helping to reduce constraints on studies of aerosols and their impacts.

## Graphical abstract

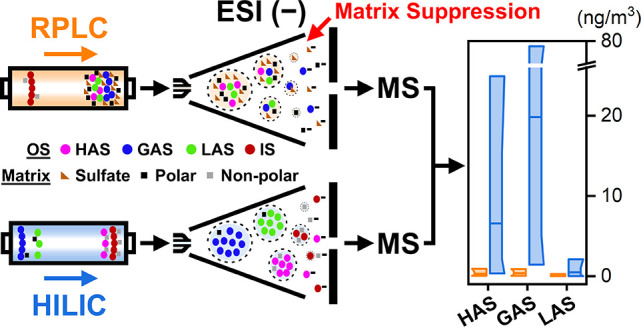

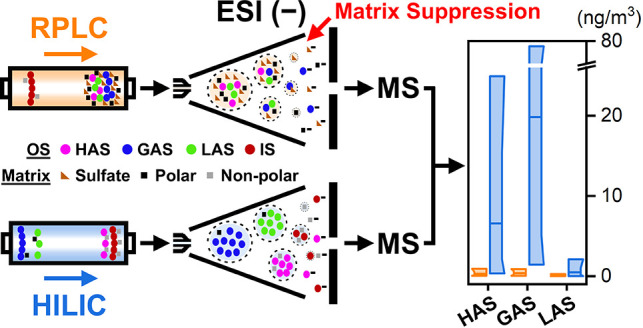

## 1 Introduction

Organosulfates (OSs) are organic compounds with a sulfate ester functionality attached to an organic residue (i.e., R-O–SO_3_H). They are ubiquitous and significant components of secondary organic aerosol (SOA) in various atmospheres (e.g., urban, rural and Arctic), posing important implications in modifying aerosol characteristics (e.g., hygroscopicity, viscosity).^1–3^ Here we focus on three multifunctional OSs containing 2–3 carbon atoms and an additional carboxyl or carbonyl functional group, abbreviated as C_2–3_OSs hereafter, including glycolic acid sulfate (GAS), lactic acid sulfate (LAS), and hydroxyacetone sulfate (HAS). They are significant contributors to the total OS abundance and are frequently reported to be among the five most abundant OSs in PM_2.5_.^4–8^ For instance, in a regional site in Beijing, China, GAS, LAS, and HAS were found to account for 47%, 11%, and 5% of the total mass of the quantified OSs (13 formulas), respectively.^8^ Their multifunctional nature enhances chemical reactivity, suggesting a potential role in redistributing aerosol speciation. The conceivable precursors of these C_2–3_OSs are glyoxal, methylglyoxal, hydroxyacetone, glycolic acid, and lactic acid,^9^ which are atmospheric oxidation products of various volatile organic compounds (VOCs), with isoprene being a major contributor. Thus, accurate quantification of C_2–3_OSs is particularly important in atmospheric environments that are strongly influenced by biogenic VOCs. Incorporating accurate concentration data of C_2–3_OSs into atmospheric models helps to achieve a refined representation of isoprene chemistry. This, in turn, advances our understanding of the complex interplays between biogenic emissions, atmospheric chemistry, and the formation of SOA.

Impeded by a lack of authentic standards, reliable quantification of these C_2–3_OSs remains elusive. Most documented detections are based on offline liquid chromatography–mass spectrometry (LC-MS) analysis, with varying instrument configurations among different laboratories.^5,6,10,11^ Electrospray ionization (ESI), operated in the negative mode, is used to monitor [M–H]^−^ ions, which are readily formed due to the acidic nature of the OS compounds. For the LC part, both reversed-phase liquid chromatography (RPLC) and hydrophilic interaction liquid chromatography (HILIC) have been applied. A survey of the literature indicates a prevalent adoption of RPLC over HILIC by atmospheric researchers (Figure S1). Given the small and polar nature of C_2–3_OSs, RPLC, employing a nonpolar or weakly polar stationary phase, is incapable of adequately retaining and resolving these compounds, making it an unsuitable choice. The widespread use of RPLC over HILIC in the atmospheric research community may stem from the limited availability, generally higher costs, and narrower column selection of HILIC columns compared to the more accessible and diverse RPLC columns. Additionally, researchers may favor RPLC to achieve multianalyte analysis in a single LC-MS run, especially when dealing with many other analytes of interest, including more hydrophobic OSs or/and non-OS species.

Choosing an inappropriate LC methodology can result in pronounced matrix effects, leading to significant measurement bias when quantifying analytes that elute in the early chromatographic region, especially when analyzing complex environmental samples. Notably, the evaluation of matrix effects for multifunctional C_2–3_OSs using RPLC-MS methods has been neglected, despite their extensive use. In this work, taking advantage of our expertise in synthesizing multifunctional organosulfates,^12–14^ we synthesized authentic standards for three multifunctional C_2–3_OSs (i.e., GAS, HAS, and LAS). Subsequently, we compared the analytical performance of two LC columns—one RPLC and one HILIC column—in quantifying the three C_2–3_OSs with negative ESI-MS. Our objectives were to quantify the measurement biases induced by matrix effects in the RPLC-MS approach, to investigate factors that contribute to matrix effects, and to establish the efficacy of the HILIC-MS method in quantifying multifunctional C_2–3_OSs.

## 2 Materials and Methods

### 2.1 Synthesis of C_2–3_OS Standards

HAS, GAS and LAS (see Table 1 for their chemical structures) were synthesized in-house for use as analytical standards. A simplified synthesis reaction strategy is presented in Scheme S1, and the detailed synthesis procedures are described in Wang et al.^9^ The synthesized standards contained mass fractions of HAS, GAS and LAS at 13.3% ± 0.6%, 11.3% ± 0.2% and 3.75% ± 0.04%, respectively, as determined by ^1^H NMR using dibromomethane as an internal standard (Text S1). The remaining components primarily consisted of residual triethylamine (Et_3_N, dominant component) and dimethylformamide from the reaction process. While achieving higher purity in the synthesized standards remains a desirable goal, it is currently not feasible due to the lack of effective purification and monitoring methods.

**Table 1:**
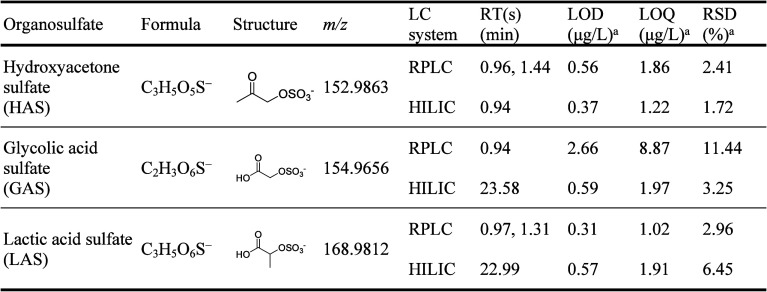
Properties of Three OS Standards Characterized by RPLC/HILIC Coupled with ESI(−)-Orbitrap MS, Including Retention Time (RT), Limit of Detection (LOD), Limit of Quantification (LOQ), and Relative Standard Deviation (RSD) of Replicate Injections

a Obtained from seven injections of 5 μg/L standard solution.

### 2.2 Sample Collection and Pretreatment

Ambient aerosol samples were collected at four sites in the Greater Bay Area in southern China. A total of 84 ambient samples and 16 field blank samples were collected and analyzed in this work, spanning two summer months (mid-July to mid-September 2020) and two months in autumn and winter (November to December 2020). The four sites are one urban site (Tsuen Wan) and one suburban site (Clear Water Bay) in Hong Kong (22°28′ N, 114°17′ E), one urban site, and one suburban site (Nansha) in Guangzhou (23°07′ N, 113°15′ E). PM_2.5_ was sampled on prebaked quartz fiber filters (20 cm × 25 cm) using high-volume samplers, each sampling period spanning 24 h from midnight to midnight. Field blanks were collected once a month. The sampled filters were wrapped with aluminum foil and stored in a freezer at −20 °C until analysis.

Sample pretreatment follows the procedure outlined by Wang et al.^12^ and a flowchart illustration is shown in Figure S3. Under the RPLC protocol, a portion of the filter samples (∼30 cm^2^) was extracted three times, each for 30 min, with 3, 2, and 1 mL of methanol (MeOH) containing saturated ethylenediaminetetraacetic acid (EDTA) in an ice-cooled ultrasonic bath. The extracts were filtered through PTFE syringe filters (0.2 μm pore size, Pall Life Sciences), transferred and combined in a conical vial, and evaporated to near dryness under an ultrapure N_2_ stream. Each extract was reconstituted in 60 μL 1:1 (v/v) MeOH/water containing 1000 ppb internal standard (IS), D17-octyl sulfate (D17-OS). Subsequently, the solution was centrifuged for 15 min to remove any remaining undissolved particles, and the supernatant was submitted to LC-MS analysis. The sample preparation steps for the HILIC protocol mirrored those of the RPLC protocol, except that the extraction solvent was 95:5 (v/v) ACN/H_2_O (yielding comparable recovery to methanol; see Table S1) and the final solution was reconstituted in 60 μL 95:5 (v/v) ACN/H_2_O that contained a mixture of ISs comprising D17-OS, camphorsulfonic acid (CPS), and ketopinic acid (KTA), each at 1000 ppb.

### 2.3 LC–MS Analysis

#### 2.3.1 LC-ESI­(−)-Orbitrap MS Analysis

C_2–3_OSs were analyzed using an Orbitrap MS (Orbitrap Exploris 120 mass spectrometer, Thermo scientific) coupled with an ultrahigh performance liquid chromatography (UHPLC) system (Dionex Ultimate 3000 Series, Thermo Scientific). Table S2 summarizes the details of two sets of LC configurations (i.e., RPLC and HILIC) and their operational conditions, which are similar to those described in Wang et al.^12^ and Cui et al.^15^ Note that the specialized RPLC column with already enhanced polar retention was employed in this study. Orbitrap MS was operated in the ESI negative mode. MS data were sequentially obtained using full scan (FS) mode and data dependent MS2 mode. All analytes were detected and quantified as their deprotonated [M–H]^−^ molecules. Further details regarding the instrumental operation parameters and data processing can be found in Section S2.2. Recovery test demonstrated satisfactory recoveries (>70%) for all analytes (Table S1), and the final concentrations were corrected for their respective recovery.

#### 2.3.2 LC-Qtrap MS Analysis

We also analyzed a subset of samples using a different MS system coupled with an RPLC column to evaluate if the matrix effect is sensitive to the mass spectrometer type. The experiment was performed using an Agilent 1290 Infinity II UHPLC system coupled with a triple-quadrupole Qtrap MS (QTRAP 4500, AB Sciex, USA) designed for LC-MS/MS analysis. The LC was operated in the same manner as described in Table S2. For MS data acquisition, ionization was achieved by ESI operating in negative mode, while detection was performed using the multiple reaction monitoring (MRM) mode. The detailed operational parameters and data analysis with this analytical system are provided in Table S3 and Section S2.3.

## 3 Results and Discussion

### 3.1 Chromatographic Characteristics of C_2–3_OSs by RPLC and HILIC Methods

Three C_2–3_OS standards were characterized by both RPLC and HILIC columns interfaced with the Orbitrap MS. Figure 1 compares the extracted ion chromatograms (EICs) monitored at the respective *m*/*z* of each analyte’s deprotonated molecule between the RPLC and HILIC methods for a standard mixture, a low PM_2.5_, and a high PM_2.5_ sample. Herein, we defined a low or high-PM_2.5_ sample as one that had levels of PM_2.5_ mass lower than or higher than 30 μg/m^3^. In high PM_2.5_ samples, concentration levels of C_2–3_OSs are also high (>80 ng/m^3^), as these two variables were positively correlated (*R* > 0.5).

**Figure 1:**
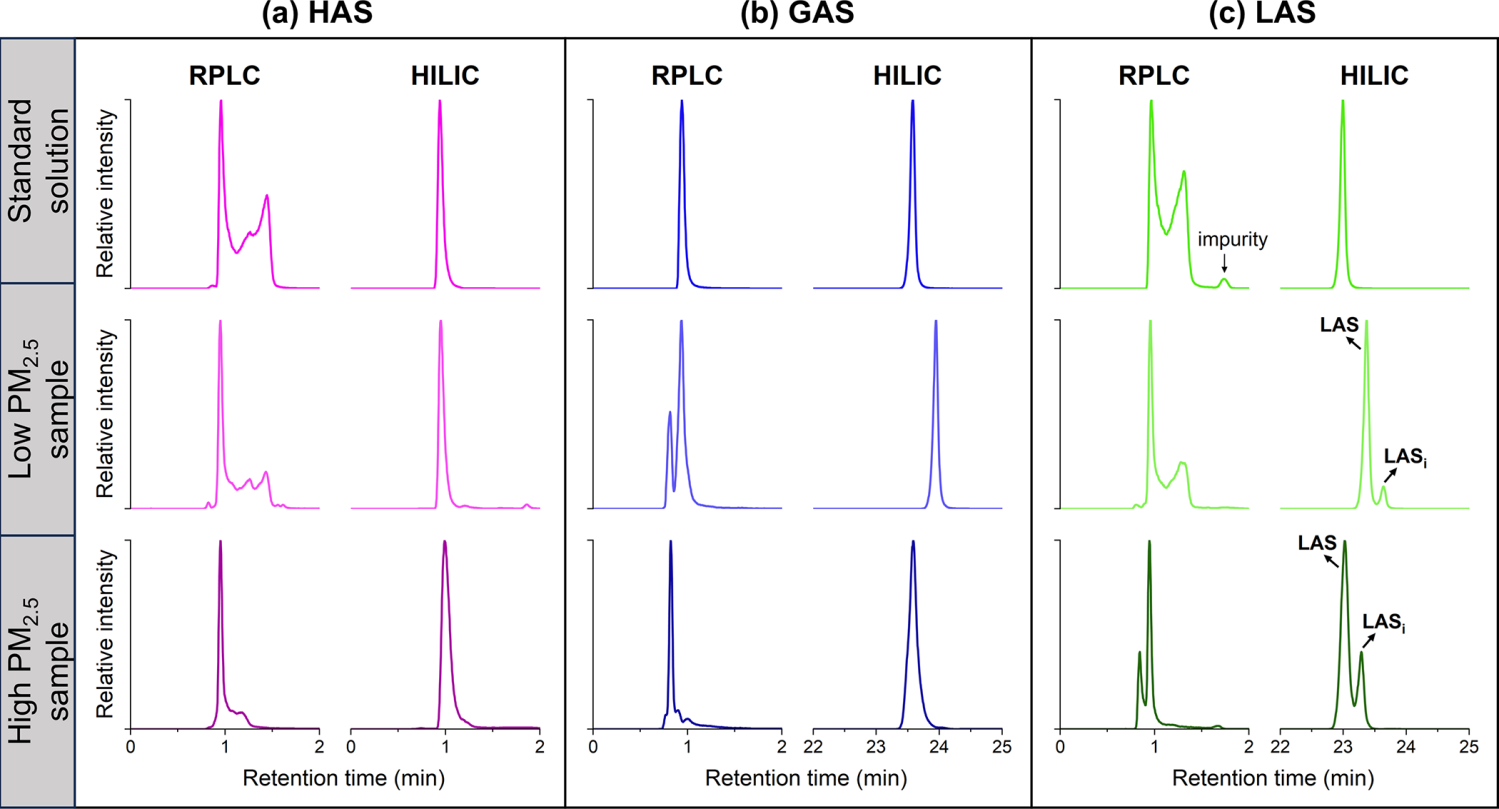
Comparison of EICs obtained with RPLC and HILIC methods for deprotonated C_2–3_OSs, including (a) HAS, (b) GAS, and (c) LAS. For each OS, three types of EICs are shown, corresponding to three different matrices with the top row for a standard solution matrix, the middle row for a low PM_2.5_ sample matrix, and the bottom row for a high PM_2.5_ sample matrix.

#### 3.1.1 Chromatographic Characteristics of the C_2–3_OS Standards

With the RPLC column, all three C_2–3_OSs coeluted within the first two minutes and the chromatographic performance was compromised, evident by the broadening and splitting of peaks for HAS and LAS. This distortion arose from the solvent effect: the injection solvent (50:50 MeOH/H_2_O) exceeded the initial mobile phase strength (1:99 MeOH/H_2_O), disrupting analyte partitioning and accelerating elution.^16,17^ GAS, being the most hydrophilic, eluted unaffected near the solvent front. Additional discussion of solvent effect can be found in Section S2.4. It is important to note that the issue of distorted peak shapes is innate to the RPLC method due to the difficulty in balancing the necessity of initiating gradient elution with a mobile phase rich in water content and the need for a stronger solvent to fully dissolve other nonpolar compounds of interest.

In contrast, HILIC resolved the three C_2–3_OSs with symmetric peaks. Although the RT of HAS (0.9 min) remained low, GAS (23.6 min) and LAS (23.0 min) exhibited significantly higher retention and enhanced separation. These improved retention properties can be attributed to the presence of a carboxylic acid moiety that enhances the analytes’ hydrophilicity and strengthens the interactions between the analytes and the stationary phase via hydrogen bonding and dipole interactions, thereby shifting the partition equilibrium toward the stationary phase.^10,18–20^ Additionally, the basic (pH 9) buffered mobile phase likely further enhances retention by sufficiently ionizing the carboxylic acid groups (thereby increasing hydrophilicity), while the buffer helps to mitigate the electrostatic repulsion between the negatively charged analytes and the stationary phase under basic conditions.^19^

#### 3.1.2 Chromatographic Characteristics of Ambient PM_2.5_ Samples

HAS, GAS, and LAS were all positively identified in ambient PM_2.5_ samples through comparison of RTs and MS/MS spectra (Figure S5). HILIC analysis maintained consistent chromatographic performance across standards and samples, with only minor RT shifts (Figure 1, HILIC panel) attributable to batch-to-batch variations in mobile phase conditions (e.g., pH value).^18,21^ Furthermore, an isomer of LAS (LAS_i_) was resolved (Figure 1c) and it will be discussed in detail in Section 3.5. RPLC analysis, however, showed poor reproducibility, particularly for GAS and LAS, both between standards and samples, as well as across different samples (note that the RPLC panel data represents a standard solution and two selected samples prepared and analyzed in the same batch). Earlier studies have similarly documented the elution of multiple peaks for GAS and LAS in aerosol samples,^22–24^ with some cautioning about potential misinterpretation in peak assignments.^23^ In our study, after careful evaluation of MS/MS data and considering the absence of other significant peaks in the EIC obtained from HILIC-MS analysis, we attributed the peak cluster eluting in the first 1.5 min window to our target analytes and LAS_i_. The phenomenon of variable RTs and distorted peak shapes was not apparent for compounds eluting at later stages, indicating that this issue was more likely to occur for poorly retained compounds and was structure dependent. For example, HAS exhibited a less impaired LC behavior compared to the other two C_2–3_OSs.

In RPLC analysis, we also noticed a trend in which the RT centroids of all C_2–3_OSs shifted toward shorter times with increasing collected PM_2.5_ mass on the filter samples (Figure 1, RPLC panel). This peculiar chromatographic behavior has been reported in a study by Fang et al., who demonstrated that matrix effects could significantly alter the peak shape and RT of bile acids when separated by a C18 column in urine samples.^25^ Building upon Fang’s findings, it is plausible that matrix components present in environmental samples, especially nonretentive inorganic ions and other small polar molecules with similar RTs, may strongly interfere with the elution and ionization of analytes by loosely binding to them. This interference can lead to variable RTs and peak shapes, thereby increasing the likelihood of errors in identification and quantification. Another potential contributing factor could be the altered pH of the mobile phase within the column due to the presence of coeluted matrix components.^25^

### 3.2 Quantification of C_2–3_OSs in Ambient Samples

#### 3.2.1 Selection of Internal Standards

In the RPLC-MS analysis of PM_2.5_-bound OSs, an IS frequently chosen in previous studies was D17-OS, due to its nonendogenous nature, possession of a sulfate functional group, and its similarity in carbon number to certain OSs (e.g., monoterpene-derived OSs).^6,12^ However, the substantially longer carbon chain of D17-OS resulted in a much higher RT compared to C_2–3_OSs. This characteristic makes D17-OS a suitable IS for OSs with higher-carbon-numbers and thus longer RTs, but ineffective in compensating for matrix effects occurring at the solvent front, which constitutes the most problematic chromatographic region. This region exhibits appreciably higher total ion abundance than other areas, as shown in Figure S6.

In studies focusing on polar OSs using HILIC, researchers commonly rely on the external standard (ES) method for quantification and seldom incorporate ISs.^3,4,10,26^ In our investigation, we experimented with three ISs, namely D17-OS, CPS, and KTA, with their LC-MS properties summarized in Table S4. In contrast to RPLC applications, D17-OS exhibited minimal retention on the HILIC column and eluted in the front apolar bulk region. This elution property made it effective for standardizing signals of HAS, which also eluted in this region of maximal matrix effects evidenced by the total ion chromatogram (Figure S6b). CPS, previously used as a surrogate standard in the absence of an authentic OS standard,^12^ exhibited irregular LC-MS behaviors in one-sixth ambient samples, rendering it unsuitable as an IS for HILIC-MS analysis (detailed in Section S3). On the other hand, KTA shares a similar carbon skeleton with CPS and contains a carboxylic group tail. While our preliminary analysis revealed that KTA naturally occurred at very low concentrations in PM_2.5_ samples, it eluted sufficiently further from the front-end and closer to GAS and LAS in the chromatogram (Figure S6b), making it a viable IS candidate for GAS and LAS.

#### 3.2.2 Calibration Methods

RPLC-MS quantification relied on IS-based calibration (D17-OS), with quadratic calibration curves (range: 5–10,000 μg/L, *R* ^2^ > 0.998) used in a few samples where peak area (PA) ratio data fell outside the linear range. However, in HILIC-MS measurements, we found that most of the PA ratios significantly exceeded the linear region, indicating substantially higher analyte concentrations were derived. This posed a challenge in building a calibration curve with a sufficiently large dynamic range. We thus instead established linear and quadratic calibration curves using the ES method, which was able to cover all measurable responses.

To evaluate the most suitable calibration methodologies for HILIC-MS analysis (IS-based or ES-based), we conducted comparative analyses using ten appropriately diluted samples with varying C_2–3_OS concentrations. We performed the standard addition (SA) method (under RPLC protocol) for each analyte to obtain unbiased reference value for method validation. To avoid excessive sample consumption, we selected five representative samples spanning high, medium, and low concentration ranges to derive a three-level calibration for each. Following comparative results (detailed in Section S4), we adopted the IS method for HAS and the ES method for GAS and LAS, with appropriate normalization, to quantify all environmental samples.

Upon comparing the results obtained from the SA method, we observed that the HILIC-MS method provided accurate measurements close to the true values, whereas the RPLC-MS method significantly underestimated the concentrations (Figure S11). The reduced signal intensity in the RPLC-MS method could be explained by the matrix suppression in the front polar bulk of RPLC, where a multitude of interfering species, including other hydrophilic OS species, small organic acids, polyols, and abundant inorganic salts extracted from the ambient samples, could coelute with our analytes within the first two minutes of the chromatographic run.^10,22^ This concurrent elution of matrix components could lead to ion suppression through several mechanisms, such as competition with C_2–3_OSs for the available charges in the ESI chamber and reaction with the precursor ions at the LC-MS interfaces, thus weakening the ionization efficiency and detection intensity of the analytes.

### 3.3 Comparison of Quantitative Results between RPLC and HILIC

Figure 2 provides an overview of the concentration data obtained using the RPLC and HILIC protocols. To facilitate a more accurate comparison, the LAS concentrations derived from HILIC-MS in this figure represent combined values for LAS and LAS_i_ (see Section 3.5). Both methods identified GAS as the most abundant C_2–3_OS, followed by HAS and LAS, a finding in alignment with previously reported measurements (Figure S1). However, a large discrepancy in absolute concentration values is evident between the two measurement approaches, with the HILIC method derived results significantly higher than those by the RPLC method by 1–2 orders of magnitude. On a sample-by-sample basis (Figure 2b), RPLC consistently yielded lower concentrations than HILIC for all analytes, with the exception of a few low-level LAS data, possibly attributable to the increased measurement uncertainties with very low particle loading (PM_2.5_ mass <10 μg/m^3^).

**Figure 2:**
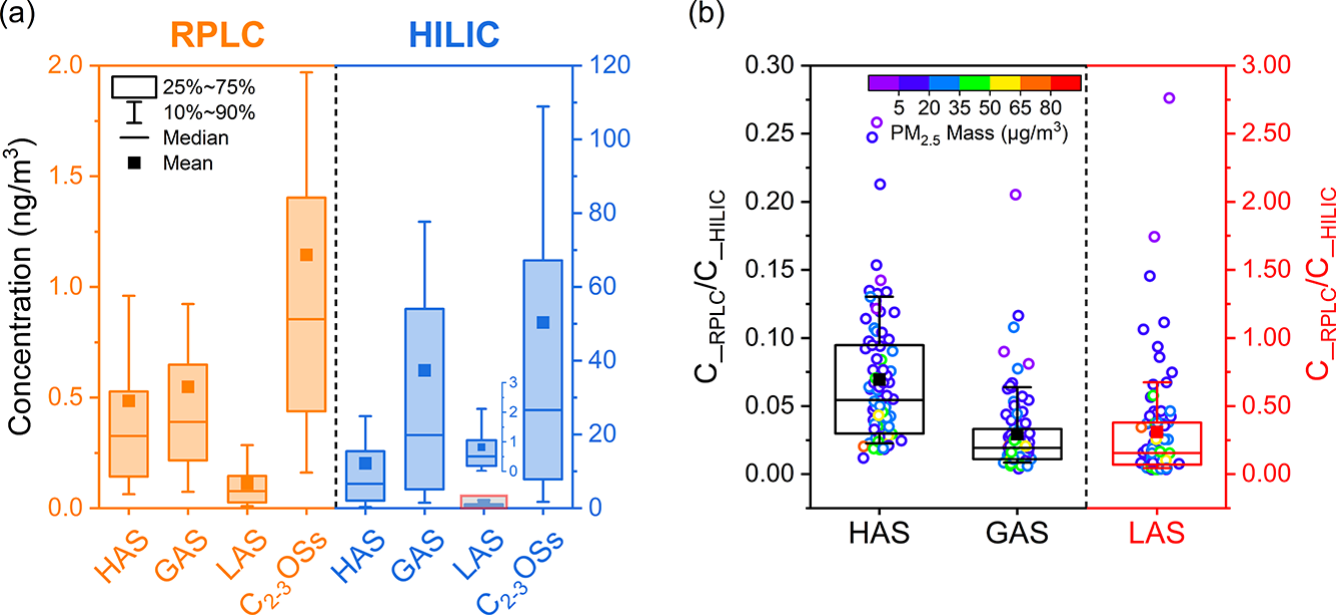
Comparison of RPLC and HILIC measurements of HAS, GAS, and HAS in 84 ambient PM_2.5_ samples: (a) concentration distributions displayed in box plots; (b) concentration ratios by the RPLC and HILIC method for HAS (left axis), GAS (left axis), and LAS (right axis). Outliers detected by Grubbs test are excluded.

The evidence supporting the influence of matrix effects was further strengthened by Figure S12, which shows negative correlations between C__RPLC_/C__HILIC_ ratio and both PM_2.5_ mass and sulfate ion (major component of PM_2.5_). These correlations suggest the involvement of sulfate and potentially other PM_2.5_ components in diminishing the MS signal of C_2–3_OSs. Additionally, a semiquantitative assessment of matrix effects indicated that higher PM-loaded samples were associated with stronger matrix suppression, as discussed in Section S5.

When employing RPLC, the GAS results showed the largest negative bias among the three small OS species, followed by HAS and LAS (Figure 2b and Section S5). Figure S13 provides a more quantitative evaluation of the species-specific disparities arising from the two LC approaches. Based on the regression statistics (Figure S14), the HILIC-measured concentrations of HAS, GAS, LAS, and total C_2–3_OSs were approximately 27-fold, 70-fold, 7-fold and 47-fold greater, respectively, than those determined by RPLC.

Notably, the unbiased HILIC-Orbitrap MS protocol quantified peak C_2–3_OSs concentration of 517 ng/m^3^ (2.3% OA, 0.9% PM_2.5_ mass) during a summer typhoon-influenced high-pollution episode (Figure S24), stressing C_2–3_OSs’ nontrivial aerosol impacts. This event, driven by regional transport of biogenic and anthropogenic precursors, stagnant meteorological conditions, and elevated oxidant (O_*x*_) concentrations, created an ideal environment for rapid precursor oxidation and subsequent OS formation. Such episodes, increasingly common under climate change, underscore the necessity of robust analytical methods like HILIC-MS to accurately resolve dynamic aerosol composition and its links to atmospheric reactivity.

### 3.4 Investigation of Underestimated Measurement Bias in RPLC-MS Analysis

We conducted additional investigation to understand the underlying reasons for the underestimated RPLC-MS measurements, categorizing potential causes as internal or external based on whether they originated within or external to the sample matrix. One external factor we explored was the use of EDTA during the sample preparation process, which we found to have an insignificant impact on sample concentrations compared to the bulk coextracted PM_2.5_ matrix (Section S6.1). On the other hand, we identified two internal and two external factors that contributed to the measurement bias. One internal factor, adduct formation, is described in Section S6.3. The remaining factors are discussed below.

#### 3.4.1 Internal Factor #1: Bisulfate Ion

In several studies of quantifying C_2–3_OSs in atmospheric particles, researchers have raised concerns regarding interference from inorganic ions in sample extracts, specifically the bisulfate ion, due to its prevalence in PM_2.5_ samples and its high ionization ability to compete for the applied charge.^15,27^ In HILIC studies, where inorganic ion peaks are separated from C_2–3_OSs, the influence of these impurities could be reasonably neglected.^27,28^ However, in RPLC analysis, the separation of inorganic sulfate from many water-soluble OS is challenging due to their comparable unretentive properties, particularly when using a conventional C18 column.^23,29^ The overlap or close elution of the bisulfate ion and C_2–3_OSs is recognized, but the resulting issue of ion suppression has not been adequately appreciated due to the simplistic single-column analysis approach.

Figure 3 illustrates superimposed chromatograms from a field sample obtained using two columns, showing base peak chromatograms, EICs of deprotonated analytes, and EICs of bisulfate clusters (detected as HSO_4_^–^ *m*/*z* 97, H_3_S_2_O_8_^–^ (H_2_SO_4_·HSO_4_^–^) *m*/*z* 195 and NaH_2_S_2_O_8_^–^ *m*/*z* 217). Under HILIC conditions, all C_2–3_OSs were the most prominent peaks at their respective RTs (Figure 3b1), indicating that the analytes were sufficiently ionized and detected at each LC fraction window. While the bisulfate ion interference was primarily detected in the short-RT region containing HAS’s elution, its impact was minimal due to its much lower intensity compared to HAS (Figure 3b3).

**Figure 3:**
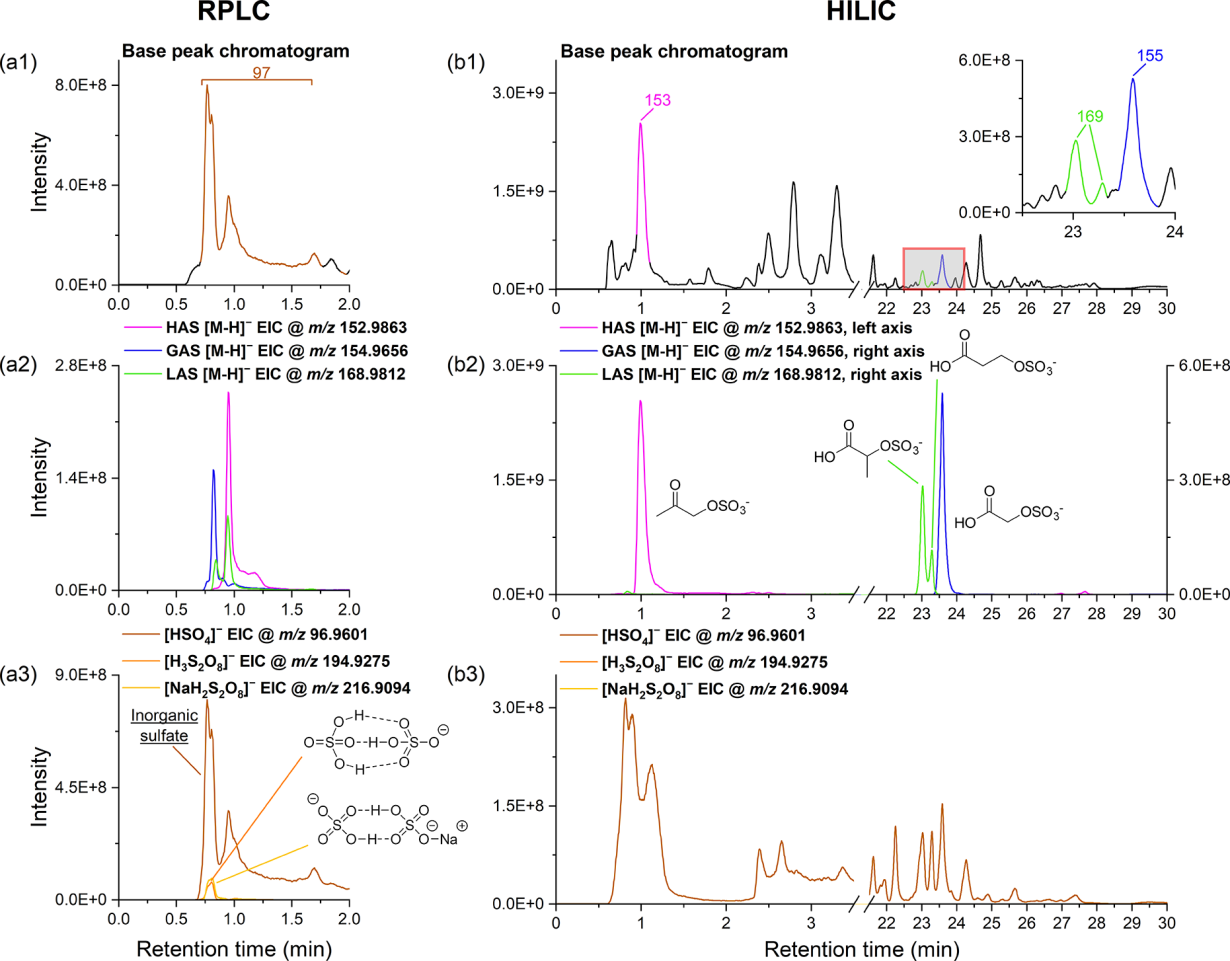
Combined chromatograms of a field sample (collected at Tsuen Wan site on 7 November 2020) obtained from the RPLC column and the HILIC column. The top panels (a1, b1) represent base peak chromatograms, and chromatographic peaks are colored with attributed [M–H]^−^ ions. The middle panels (a2, b2) represent EICs of deprotonated analytes. The bottom panels (a3, b3) represent EICs of bisulfate clusters. Note that H_3_S_2_O_8_^–^ and NaH_2_S_2_O_8_^–^ ions were not detected in the EIC by the HILIC-MS protocol.

Conversely, under RPLC conditions, a different scenario was observed. As shown in Figure 3a1, in the first 0.75–1.75 min window, the HSO_4_^–^ ion was the base peak and had a predominant presence. Unfortunately, the three C_2–3_OSs also eluted during this time frame (Figure 3a2), thus subjected to considerable interference from HSO_4_^–^ ion. The first major HSO_4_^–^ peak (RT 0.8 min), commonly attributed to the bulk sulfate or/and bisulfate ions extracted from PM_2.5_ samples,^22,29^ acts as an endogenous suppressor. The subsequent presence of HSO_4_^–^ might be generated during in-source fragmentation of various poorly retained sulfate-containing compounds. Additionally, a molecular species H_3_S_2_O_8_^–^ (H_2_SO_4_·HSO_4_^–^), a cluster ion of HSO_4_^–^, together with its sodium adduct, NaH_2_S_2_O_8_^–^, were also observed at the same RT as the first eluting HSO_4_^–^ peak (Figure 3a3). The chemical structures of these two cluster ions were proposed based on previous theoretical calculations.^30^

Notably, Figure 3a2 reveals that the sample matrix induced a shortened retention of GAS on the RPLC column, placing this species within the same elution zone where HSO_4_^–^ and its clusters primarily eluted. This resulted in more pronounced ion suppression for GAS compared to the other two analytes, explaining why GAS was most underestimated quantitatively. We note that in a study inorganic sulfate and GAS were reported to have similar yet distinguishable elution behavior and were separable.^29^ This discrepancy from our observation likely arises from variations in factors influencing the retention of early eluting species, such as the varying compositions of PM_2.5_ sample matrices (influenced by site location and nearby sources), the sample preparation process, and the LC settings. By comparison, the overlap of the HSO_4_^–^ peak with HAS and LAS was less intensive, resulting in less negative impact on the response and quantitative assessment of these analytes. The fundamental pattern of bisulfate ion’s interference in RPLC-MS analysis remained consistent across various sampling scenarios (different sites and seasons), as demonstrated in Figure S19.

Additional mass spectrometric evidence is presented in Figure S20. In the HILIC-MS analysis, C_2–3_OS deprotonated molecules dominated the mass spectra, with the bisulfate ions presenting minimal interference (Figure S20a). The consistent intensity ratio between bisulfate and OS ions suggested that HSO_4_^–^ is likely an in-source fragmentation product of the target analytes. In contrast, the RPLC-MS analysis showed mass spectra characterized by abundant HSO_4_^–^ ions, which suppressed analyte responses, particularly impacting GAS (Figure S20b). Additionally, the two HSO_4_^–^ clusters emerged as strong competitors for ionization. HAS and LAS eluted in complex chromatographic fractions containing various high-abundance masses. Among the various interferents that may have collectively masked target detection, HSO_4_^–^, likely arising from the cleavage of fragile precursor ions containing a sulfate functional group, again stood out as the main suppressor due to its prominent presence.

In sum, HILIC created a more favorable chromatographic elution environment for the detection of polar OS, enabling sufficient ionization of C_2–3_OSs with minimal interference from the HSO_4_^–^ ion. In contrast, C_2–3_OSs quantified by RPLC-MS encountered varying levels of ion suppression, with HSO_4_^–^ identified as the most crucial internal suppressor.

#### 3.4.2 External Factor #1: Application of Specific MS System

To investigate the potential impact of the MS instrument type on the underestimated results in the RPLC-MS method, we repeated the RPLC-MS analysis using an ESI(−)-Qtrap MS system. Experimental details are presented in Section S6.1. The comparative analysis revealed that RPLC-MS concentrations obtained from the two MS systems were of similar magnitude, and both were 1 order of magnitude lower than the HILIC-MS concentrations (Figure S18). This clear disparity confirms the substantial influence of matrix effects on the quantification of C_2–3_OSs when employing the RPLC protocol. Additionally, our observations showed that the MS/MS data obtained from Qtrap exhibited even greater negative bias than the data acquired from the RPLC-Orbitrap MS system (Figure S22), indicating a higher susceptibility to matrix effects. Potential causes for these observations are discussed in Section S6.4.

In addition to its susceptibility to matrix effects, the MS/MS data obtained from MRM analysis appeared to lack sufficient selectivity compared to the high-resolution FS data by the Orbitrap MS. Despite the target-specific nature of MRM, its lower resolution limits its ability to distinguish between molecules with similar masses. This limitation can lead to erroneous peak assignments when the matrix peak overlaps with or is close to the analyte peak.^31^ Figure S23 provides a compelling example demonstrating the detection of LAS using the two MS systems coupled with the same RPLC column. In the Qtrap analysis, the EIC obtained from the sample differed significantly from the spiked standard. Specifically, the traces between 1.00–1.70 min, corresponding to LAS based on the aligned RT, exhibited dissimilar peak shapes compared to the standard. Additionally, there was a prolonged detection window from 1.70 to 2.50 min. Given that matrix effects can unpredictably modify the chromatographic behavior of LAS, correct PA integration becomes even more challenging. Relying solely on the selectivity of MRM scans might lead to false positives if suspected peaks appearing near the expected RT are included without proper verification. By employing Orbitrap detector with ultrahigh mass resolving power, a second trace with a very close *m*/*z* value (169.0176 Da) to the monitored ion of LAS (168.9812 Da) was differentiated, mirroring the MRM chromatogram of the 169 → 97 transition, particularly in the latter RT region highlighted by the gray box in Figure S23. This discovery strongly suggests that at least one other endogenous impurity, eluting at a similar time, shared the same MRM pattern as LAS, thus complicating the targeted EIC. The chemical composition of the unknown *m*/*z* 169 (assumed as [M–H]^−^ precursor ion) was determined to be C_4_H_9_O_5_S^–^, based on accurate mass measurement. Given the chemical similarity (i.e., polar structure and sulfate functionality) and the availability of β-proton to generate HSO_4_^–^ product ion,^32^ possible structures for C_4_H_9_O_5_S^–^ were proposed (Figure S23). Although the interfering C_4_H_9_O_5_S^–^ trace only slightly overlapped with the analyte peak, its impact on erroneous quantification could become more pronounced as the LAS signal had already been significantly suppressed by the matrix under the analysis condition of Qtrap system.

#### 3.4.3 External Factor #2: Impurities in Synthesized Standards

The C_2–3_OS standards contained residual Et_3_N (dominant component) from the synthesis (Scheme S1), with the target OSs constituting 3.75%–13.3% of the mixtures by mass. With no side products of sulfation detected, we investigated the potential impact of the residual impurities on the response factor (i.e., the slope of calibration curve) of the analytes. Dimethylformamide exhibited minimal influence in the LC-MS analysis due to its weaker MS signal and chromatographic separation from analytes in both columns (RT = 2.8 min in RPLC; RT = 1.2 min in HILIC). Et_3_N, however, produced significant signals in all three C_2–3_OS standards. While Et_3_N could be well separated from the C_2–3_OS peaks on the HILIC column, it coeluted with C_2–3_OSs on the RPLC column (Figure S2). In ESI, this impurity, being basic, can act as a proton acceptor and promote the formation of negatively charged ions by neutralizing their acidic protons. Consequently, this process enhances the ionization efficiency of the acidic sulfate ester group, potentially leading to an overestimated response factor for C_2–3_OS when using the RPLC protocol. This enhancement effect was not observed to the same extent in samples lacking dominant basic impurities. Consequently, analyte concentrations in such samples may be underestimated, since the calibration curve—based on artificially elevated response—does not accurately reflect the true ionization behavior under normal ionization conditions. Since further purification of the synthesized products is impractical,^9^ the reliability of the response factor obtained through the RPLC-MS method is brought into question. This finding introduces additional uncertainty to the previous RPLC measurements obtained using calibration curves based on synthesized standards that were not completely pure.

### 3.5 Revelation of Novel Multifunctional C_2–3_OSs Using HILIC-MS Method

In the HILIC method, the EIC of LAS in the PM_2.5_ samples (Figure 1c, middle and bottom panels) revealed two closely eluted peaks. The taller peak on the left was confirmed to be LAS through comparison with the standard. The smaller peak on the right was identified as an isomer of LAS, which we denoted as LAS_i_. The close RT and analogous MS/MS spectra suggest that LAS_i_ bears structural similarity to LAS and likely contains a carboxyl moiety. Given LAS’s single chiral center, LAS_i_ was determined not to be a diastereomer of LAS. An enantiomer was also ruled out as enantiomers would exhibit identical fragmentation patterns. Notably, the consistent presence of *m*/*z* 59.0139 (C_2_H_3_O_2_^–^) in LAS_i_’s MS/MS spectra (Figure S5d) suggested a heterolytic cleavage of a CH_2_O group from the primary fragmented product, *m*/*z* 89.0233 (C_3_H_5_O_3_^–^). Consequently, the structure of the product ion from loss of neutral SO_3_ (i.e., C_3_H_5_O_3_^–^) should bear a CH_2_O group at the chain end. Based on these deductions, LAS_i_ was proposed to be 2-carboxyethyl sulfate, i.e., the sulfate ester of 3-hydroxypropanoic acid or hydracrylic acid sulfate (Figure S5d). To our knowledge, this study marks the first resolution and tentative assignment of a chemical structure to this compound. However, in RPLC-MS analysis, identifying LAS_i_ was challenging due to its unpredictable retention behavior and the infrequent collection of MS/MS data owing to its low abundance (Figure 1c).

By using LAS’s response factor for estimation, the ambient abundance of LAS_i_ was determined to be at subng/m^3^ levels, lower than that of LAS, which fell in the low ng/m^3^ range (Figure S25a). The strong correlation observed between this pair of isomers (Figure S25b) suggests a common origin for LAS_i_ and LAS.

Expanding beyond the discovery of LAS_i_, the HILIC-MS method facilitated the detection and separation of several other multifunctional C_2–3_OSs (Figure 4). We confirmed the presence of two glycerol sulfate (GcS) isomers, one glyceric acid sulfate (GcAS) isomer, one ethylene glycol sulfate (EGS) isomer and two hydroxypropylsulfate (HS) isomers, which were previously identified in field studies but received limited recognition.^3,26^ Notably, to the best of our knowledge, we also identified several novel isomers/species, including one additional GcAS isomer, one HS isomer, one EGS isomer, hydroxypropanesulfonic acid sulfate (HPAS) and acrylic acid sulfate (AAS). The assignment of these compounds was based on careful evaluation of their MS2 spectra (e.g., HSO_4_^–^ ion as the most abundant fragment) and the RT plausibility (e.g., carboxylic functionality delaying elution). Proposed candidate structures are presented in Figure 4a. The success in comprehensive identification and separation of isomers was largely attributable to the superior isomeric separation capability of HILIC compared to RPLC, where isomeric mixtures encountered severe elution overlaps and displayed unfavorable peak shapes (Figure 4a). Moreover, as observed in three representative samples, matrix effects in RPLC-MS also led to significant underestimation of the atmospheric abundance of these species (using GAS as surrogate standard). The two GcAS isomers correlated strongly with each other and with methylglyceric acid sulfate (Figure S26), suggesting shared biogenic origins via isoprene oxidation and potential utility as molecular tracers. Beyond multifunctional C_2–3_OSs, HILIC-MS also demonstrated exceptional ability in resolving other alkyl C_2–3_OSs (e.g., ethyl/propyl sulfates, Figure S27), underscoring its versatility.

**Figure 4:**
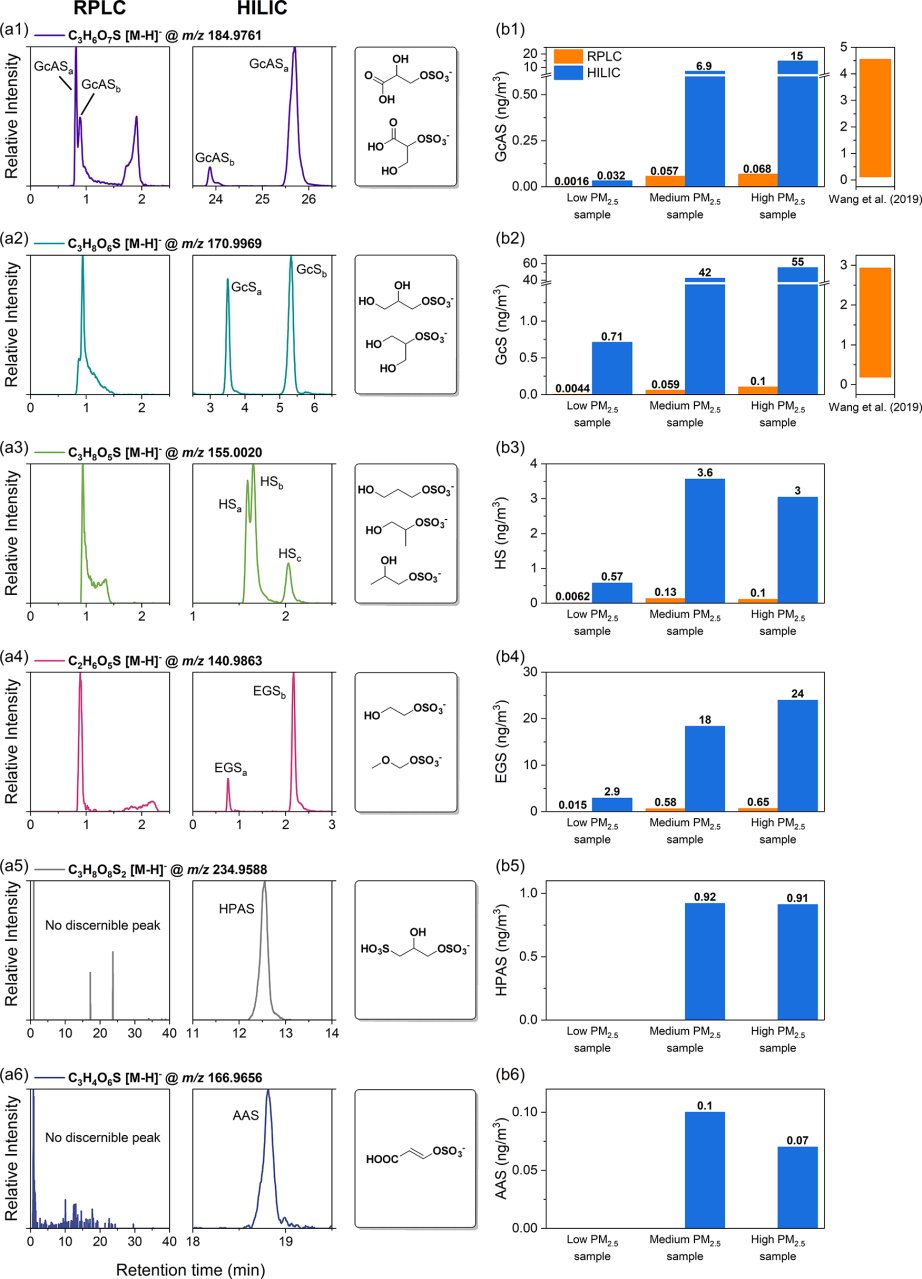
Comparison of RPLC-MS and HILIC-MS methods for (a) identification and (b) semiquantification of GcAS, GcS, HS, EGS, HPAS, and AAS in this study and Wang et al.^6^

These findings strengthen the significant benefits of HILIC separation in studying small polar compounds like C_2–3_OSs in environmental samples. The method enhances the likelihood of revealing previously unidentified species and ensures accurate quantification of individual compounds. This analytical approach provides a more detailed picture of OS composition in atmospheric samples, contributing to improved understanding of their sources, formation mechanisms, and environmental impacts.

## 4 Environmental Implications

Conventional analytical workflows employing RPLC-MS method systematically underestimate multifunctional C_2–3_OSs in atmospheric aerosols due to unresolved matrix effects and methodological limitations. This bias can propagate into climate and air quality models, leading to misrepresentation of OS abundance and their role in SOA formation, and obscuring evaluation of critical feedback between biogenic emissions, aerosol chemistry, and climate. In contrast, HILIC circumvents these challenges due to its intrinsic suitability for polar compounds, enabling accurate quantification of polar OSs and separation of previously undetected isomers on the chromatographic column. By demonstrating the capturing of dynamic aerosol composition—such as C_2–3_OSs concentration up to 517 ng/m^3^ during extreme pollution episodes—we show that the HILIC-MS method could assist refined source apportionment and advance mechanistic understanding of SOA formation under real-world conditions.

Beyond C_2–3_OSs, this work advocates HILIC as a gold-standard approach for analyzing other small, polar analytes in the complex PM_2.5_ extract matrices (e.g., organic acids, sugars, oxidation tracers), which are similarly vulnerable to matrix suppression in RPLC. Our findings urge the atmospheric community to (1) re-evaluate historical data sets of polar compounds obtained using RPLC, and (2) prioritize separation techniques tailored to analyte polarity and use appropriate IS(s) when designing speciation studies. These methodological improvements are critical to generating reliable, policy-relevant data on aerosol composition, particularly given the significant uncertainties surrounding SOA in current climate models.

## Supplementary Material



## Notes

Additional experimental details; supplemental chromatograms and MS/MS spectra; details of internal standards and calibration methodologies; and supplementary assessment and investigation of matrix effects (PDF)
