# Supplementary material for: Accurate Quantification of Multifunctional C2–3 Organosulfates in Atmospheric Aerosols Using Liquid Chromatography-Electrospray Ionization Mass Spectrometry: Overcoming Matrix Effects and Underestimation
Source: Environ Sci Technol. 2025 Jun 17;59(25):12812–21. doi: 10.1021/acs.est.5c01846 (PMC12224290; doi:10.1021/acs.est.5c01846)
Supplement: Supplementary file 1 [file es5c01846_si_001.pdf]

Supporting information

Accurate Quantification of Multifunctional $C_{2–3}$ Organosulfates in Atmospheric Aerosols Using Liquid Chromatography-Electrospray Ionization Mass Spectrometry: Overcoming Matrix effects and Underestimation

Shumin Liang[1], Yuchen Wang[2], Hanzhe Chen[3], Wan Chan[1], Jian Zhen Yu[1,3]*

[1] Department of Chemistry, The Hong Kong University of Science and Technology, Kowloon 999077, China

[2] College of Environmental Science and Engineering, Hunan University, Hunan 410082, China

[3] Division of Environment and Sustainability, The Hong Kong University of Science and Technology, Kowloon 999077, China

*Corresponding author: jian.yu@ust.hk

Number of Pages	29
Number of Sections	7
Number of Reaction Scheme	1
Number of Figures	27
Number of Tables	4

Table of Contents

Figure S1. Atmospheric measurements (average value) of particulate C_{2-3}OSs in the literature..........4

S1. Synthesis of C_{2-3}OS standards. 4

Scheme S1. Reaction strategy for synthesizing C_{2-3}OSs..4

Figure S2. Detection of Et_3N, a residual reagent, in the three synthesized OS standards. (a1), (b1), and (c1) show the RPLC EICs for HAS, GAS, and LAS standards while (a2), (b2), and (c2) show the RPLC EICs for Et_3N co-eluting in the synthesized standard HAS, GAS, and LAS, respectively. Similarly, (A1), (B1), and (C1) show the HILIC EICs for HAS, GAS, and LAS standards while (A2), (B2), and (C2) show the HILIC EICs for Et_3N co-existing in the synthesized standard HAS, GAS, and LAS, respectively. 6

S2. Sample extraction and LC-MS analysis..7

Figure S3. Flow chart of the experimental section. 7

Table S1. Recovery rates of C_{2-3}OSs and a surrogate standard. 7

Table S2. LC configurations and operational conditions..8

Table S3. Optimized MS parameters for MRM transitions using LC-Qtrap-MS. 9

Figure S4. Comparisons of RPLC EICs of HAS, GAS and LAS standards prepared in three different solvents, including 50:50 MeOH/H2O (left column), 100% H_2O (middle column), and 100% MeOH (right column)..10

Figure S5. MS/MS spectra and proposed fragmentation pathways of C_{2-3}OSs detected in $PM_{2.5}$ samples. 11

S3. Supplementary information on internal standards 11

Figure S6. Total ion chromatograms of one demonstration sample obtained from (a) RPLC and (b) HILIC systems. Elution positions of C_{2-3}OSs and ISs (underlined) are indicated with arrows. 12

Table S4. Properties of ISs used in this study. 12

Figure S7. Comparison of EICs of deprotonated CPS obtained with HILIC-Orbitrap MS under different sample conditions. 13

S4. Comparative analyses of different calibration methodologies..13

Figure S8. Example calibration curves for three C_{2-3}OSs using linear (dashed line) and quadratic (solid line) regression. Panels (a1-3) show IS calibration in RPLC-MS and panels (b1-3) show IS calibration in HILIC-MS analysis. Panels (c1-3) show ES calibration in HILIC-MS analysis. Panels (d1-3) show SA in RPLC-MS analysis. 14

Figure S9. Comparison of the calibration curves for C_{2-3}OSs in standard solution (1:1 MeOH/H_2O) and sample matrices in the RPLC-MS method. To facilitate comparison, all PA have been offset by the lowest PA in each curve 15

Figure S10. Comparison of OS concentrations generated with different calibration approaches for one demonstration sample. Asterisk-marked approach was used for sample calculations in the HILIC-MS analysis. 15

Figure S11. Scatter plots of concentrations determined by (a) RPLC and (b) HILIC methods vs those determined by the SA method. The dotted line represents the 1:1 line of perfect agreement. 16

S5. Assessing matrix effects in environmental samples 17

Figure S12. Scatter plots of $C_{\_RPLC}/C_{\_HILIC}$ ratio vs $PM_{2.5}$ mass (left panel) and sulfate ion (right panel). 17

Figure S13. Comparison of results obtained by different methods for C_{2-3}OSs in (a) high concentration samples, (b) medium concentration samples, and (c) low concentration sample. 18

Figure S14. Correlations between HILIC and RPLC measurements for (a) HAS, (b) GAS, (c) LAS and (d) total $C_{2–3}$OSs. NS: Nansha; GZ: urban Guangzhou; WB: Clear Water Bay; TW: Tsuen Wan. 18

S6. Investigation of the underestimated measurement bias 18

Figure S15. Simplified flow chart of the supplementary experiments. 19

Figure S16. Comparison of recovery of $C_{2–3}$OSs spiked on blank filters that were extracted using different media (methanol vs methanol saturated with EDTA) and determined by three different combinations of LC and MS configurations........20

Figure S17. RPLC EICs of deprotonated HAS, GAS, LAS and EDTA obtained from the RPLC-Orbitrap MS system. 21

Figure S18. Comparisons of HAS, GAS, and LAS concentrations in three $PM_{2.5}$ samples as determined following sample extraction w/o EDTA vs w/ EDTA in the methanol solvent and measured by three LC-MS configurations. The dotted line represents the 1:1 line of perfect agreement. 22

Figure S19. RPLC chromatograms comparing bisulfate ion interference across six field samples collected from different sites (Tsuen Wan, TW; Guangzhou, GZ) and seasons (autumn, winter, summer). 22

Figure S20. MS1 profiles at the elution RTs of $C_{2–3}$OSs in (a) HILIC-MS and (b) RPLC-MS analyses of a field sample. 23

Figure S21. Detection of sodium adduct of GAS $[M-2H+Na]^-$ in $PM_{2.5}$ sample. 24

Figure S22. Scatter plot of Qtrap concentration vs Orbitrap concentration. 24

Figure S23. LAS detection by (a) RPLC-Qtrap MS and (b) RPLC-Orbitrap MS. 25

S7. Analysis of $C_{2–3}$OSs using HILIC-ESI-Orbitrap MS method 26

Figure S24. Concentration time series of $C_{2–3}$OSs and $PM_{2.5}$ at the four sites. TW: Tsuen Wan; WB: Clear Water Bay; GZ: urban Guangzhou; NS: Nansha. 26

Figure S25. Quantification of LAS and LAS_i using HILIC-MS method: (a) Overall range of concentration data; (b) Correlation of LAS and LAS_i concentrations. 26

Figure S26. Scatter plots of two GcAS isomers (left) and $GcAS_{a+b}$ vs MGS (right). 27

Figure S27. EICs of m/z values corresponding to the deprotonated ethyl sulfate ($C_2H_6O_4S$) (Top row) and propyl sulfate ($C_3H_8O_4S$) (bottom row) obtained using RPLC-MS and HILIC-MS methods. EICs showed no significant peaks outside the displayed RT ranges. 27

References 27

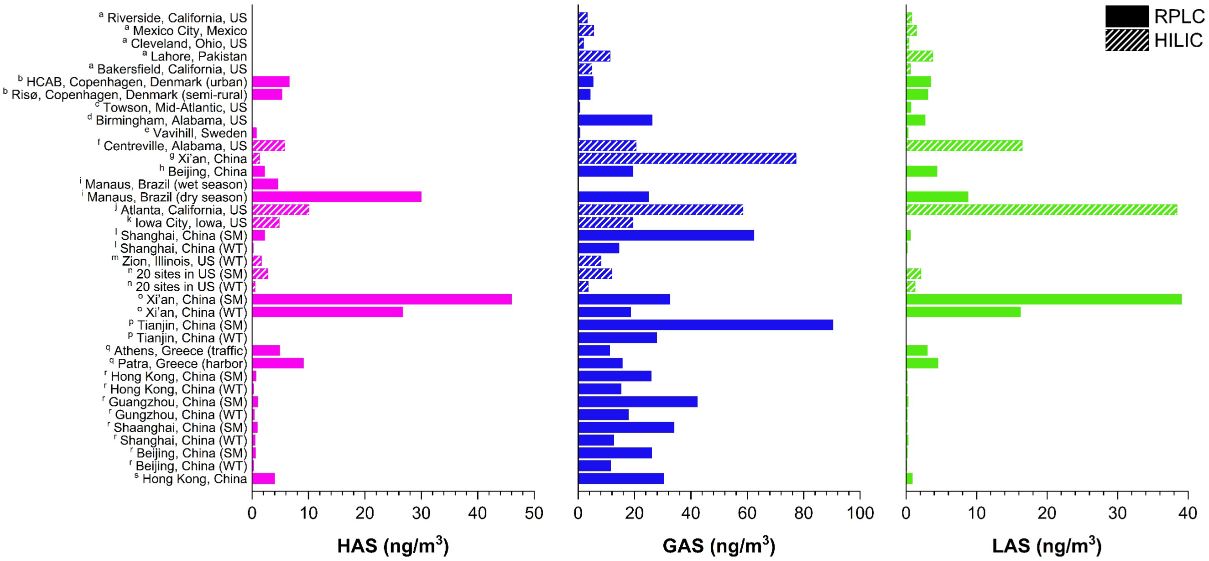

Figure S1. Atmospheric measurements (average value) of particulate C_{2-3}OSs in the literature. Data are sorted top to bottom by publication year, extracted from [a] Olson et al., 2011; [b] Nguyen et al., 2014; [c] Meade et al., 2016; [d] Rattanavaraha et al., 2016; [e] Martinsson et al., 2017; [f] Hettiyadura et al., 2017; [g] Huang et al., 2018; [h] Wang et al., 2018; [i] Glasius et al., 2018; [j] Hettiyadura et al., 2019; [k] Hughes and Stone, 2019; [l] Cai et al., 2020; [m] Hughes et al., 2021; [n] Chen et al., 2021; [o] Glasius et al., 2022; [p] Ding et al., 2022; [q] Kanellopoulos et al., 2022; [r] Wang et al., 2022, [s] Wang et al., 2023. Among the total of 19 studies,[1–19] 7 (a, f, g, j, k, m, and n) adopted HILIC-MS approach.

S1. Synthesis of C_{2-3}OS standards.

HAS, GAS and LAS were synthesized in-house for use as analytical standards. A simplified reaction strategy is presented in Scheme S1, and detailed synthesis procedures are described in Wang et al.[19]

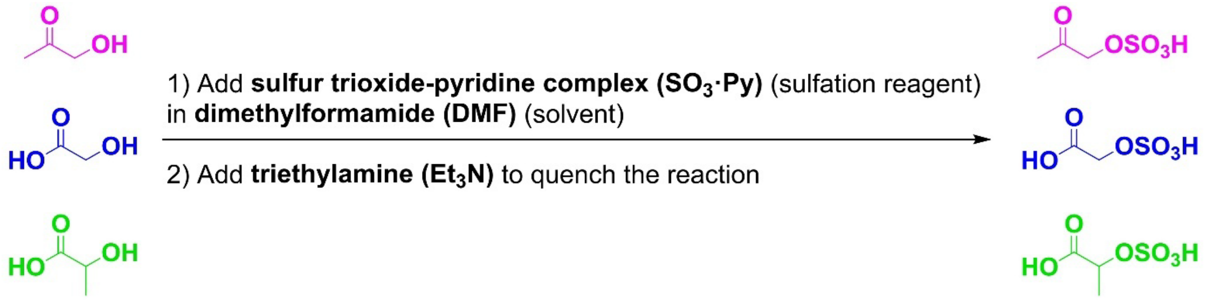

Scheme S1. Reaction strategy for synthesizing C_{2-3}OSs.

The mass fraction of the target OS in each synthesized standard sample P_x (expressed in % g/g) was determined by quantitative ^{1}H NMR using dibromomethane as the internal standard (IS) of known purity P_{IS}. The calculation was based on the direct comparison of NMR signal integrals[20–22]:

$$\frac{I_x}{N_x} \propto \frac{W_{sample} \times P_x}{M_x}$$

$$\frac{I_{IS}}{N_{IS}} \propto \frac{W_{IS} \times P_{IS}}{M_{IS}}$$

which yields:

$$P_x = \frac{I_x}{I_{IS}} \times \frac{N_{IS}}{N_x} \times \frac{M_x}{M_{IS}} \times \frac{W_{IS}}{W_{sample}} \times P_{IS}$$

where *I* is the integral area of NMR signal, *N* is the number of proton generating the signal, *M* is the molar mass (g/mol), and *W* is the weighted mass (g). This equation enables accurate determination of the analyte's mass fraction by relating the NMR integrals to the known amount and purity of the IS.

Sources of uncertainty in the determined mass fraction P_x include:

1) peak area ratio $\frac{I_x}{I_{IS}}$: 0.044% (relative uncertainty, from Schoenberger et al.[20])
2) molecular mass M_x and M_{IS}: assumed negligible
3) IS mass: ±0.0001g
4) sample mass: ±0.0001g
5) IS purity: 0.1% (relative uncertainty, from Schoenberger et al.[20])
6) systematic error (air buoyancy correction): 0.035% (relative uncertainty, from Schoenberger et al.[20])

The expanded propagated uncertainty *u* in P_x was calculated as:

$$\frac{u}{P_x} = k \times \sqrt{(0.044\%)^2 + \left(\frac{0.0001}{W_{IS}}\right)^2 + \left(\frac{0.0001}{W_{sample}}\right)^2 + (0.1\%)^2 + (0.035\%)^2}$$

where *k* is the coverage factor (*k*=2 for 95% confidence interval).

Accordingly, the mass fractions (±uncertainty) of the synthesized standards were determined to be: HAS: 13.3%±0.6%; GAS: 11.3%±0.2%; and LAS: 3.75%±0.04%.

As mentioned in Section 2.1, the synthesized standards contained residual triethylamine (Et_3N) and dimethylformamide as primary components. Figure S2 demonstrates the significant presence of Et_3N in both RPLC-MS and HILIC-MS analyses. The impact of this impurity on both analytical methods is discussed in the main text Section 3.4.3.

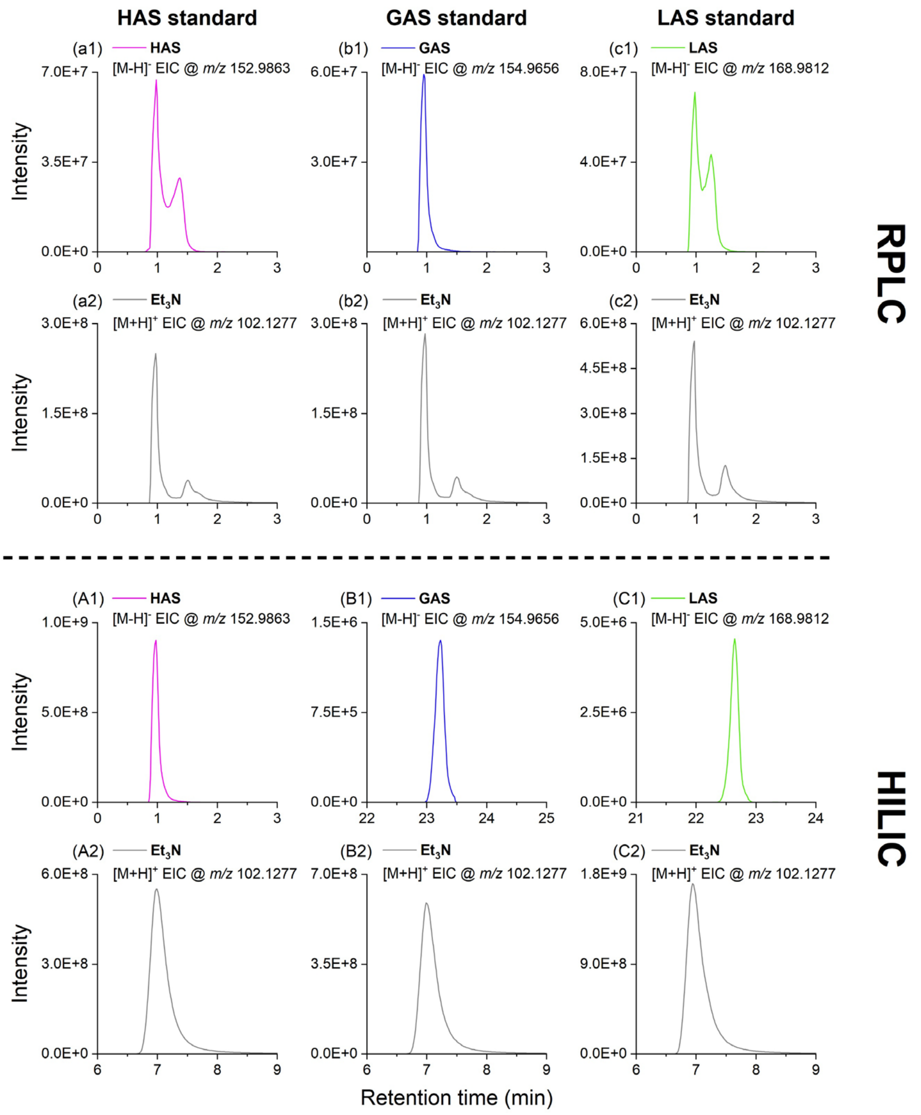

Figure S2. Detection of Et_3N, a residual reagent, in the three synthesized OS standards. (a1), (b1), and (c1) show the RPLC EICs for HAS, GAS, and LAS standards while (a2), (b2), and (c2) show the RPLC EICs for Et_3N co-eluting in the synthesized standard HAS, GAS, and LAS, respectively. Similarly, (A1), (B1), and (C1) show the HILIC EICs for HAS, GAS, and LAS standards while (A2), (B2), and (C2) show the HILIC EICs for Et_3N co-existing in the synthesized standard HAS, GAS, and LAS, respectively.

S2. Sample extraction and LC-MS analysis.

Figure S3 is a schematic diagram of the overall experimental workflow.

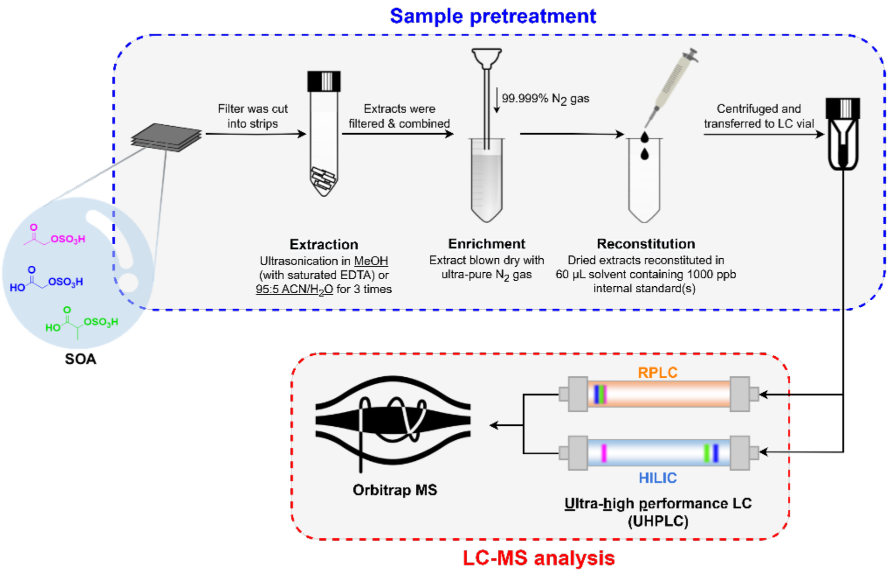

Figure S3. Flow chart of the experimental section.

S2.1 Characterization of extraction efficacy by extraction solvents

To evaluate the extractability of two solvents (i.e., MeOH and 95:5 ACN/H_2O) for $C_{2\text{–}3}$OSs and to monitor the possible degradation or chemical reactions of analytes with solvent during the ultrasonic process, we conducted recovery test to quantitatively determine the extraction efficiency. A standard mixture was prepared in water containing 500 ppb (μg/L) $C_{2\text{–}3}$OSs (i.e., HAS, GAS and LAS) and 1000 ppb CPS. CPS, which was not present in environmental samples, served to indicate any unusual loss or contamination during sample pretreatment. 50 μL aliquot of standard mixture was spiked onto the pre-baked 30 cm^2 blank quartz filter, and three replicates were performed for each solvent. It should be noted that our pilot test showed no major difference between the results of spiking a single standard and a mixed standard solution, thus we used standard mixture for simplification. Fortified filters were processed with the same procedure as the samples except that in the HILIC protocol, CPS was excluded from the final reconstitution solution. Table S1 summarizes the recovery rates of the three $C_{2\text{–}3}$OSs and CPS measured using the two LC protocols.

Table S1. Recovery rates of $C_{2\text{–}3}$OSs and a surrogate standard.

**Category**	**Compound**	**Recovery rate (%)**	
		RPLC (extracted by MeOH)	HILIC (extracted by 95:5 ACN/H_2O)
**$C_{2\text{–}3}$OSs**	Hydroxyacetone sulfate (HAS)	89.4±1.8	109.0±6.2
	Glycolic acid sulfate (GAS)	83.7±1.7	70.4±3.4
	Lactic acid sulfate (LAS)	86.3±0.7	76.4±2.9
**Surrogate**	Camphorsulfonic acid (CPS)	87.7±2.1	83.3±1.3

S2.2 LC-Orbitrap MS analysis parameters and data processing

Table S2. LC configurations and operational conditions.

LC	Column	Mobile phase	Gradient elution program	Flow rate, Injection volume & Temperature
RPLC	ACQUITY HSS T3 column (2.1×100 mm, 1.8 µm particle size, Waters) with a pre-column (1.8 µm, 2.1×5 mm)	Eluent A: water containing 0.1% acetic acid<br>Eluent B: methanol containing 0.1% acetic acid	Eluent B:<br>1% for 2.7 min, raised to 54% in 15.2 min (held for 1 min), further raised to 90% in 7.5 min (held for 0.2 min), decreased to 1% in 1.8 min (held for 11.6 min)	0.3 mL/min; 5 µL; 40 °C
HILIC	ACQUITY UPLC BEH Amide column (2.1×100 mm, 1.7 µm particle size, Waters) with a pre-column (1.7 µm, 2.1×5 mm)	Eluent A: water containing 10mM ammonium acetate (pH~9.0)<br>Eluent B: 95:5 ACN/H_2O (v/v) containing 10mM ammonium acetate (pH~9.0)	Eluent A:<br>0% for 4 min, raised to 15% in 16 min (held for 4 min), decreased to 0% in 1 min (held for 5 min)	0.3 mL/min; 5 µL; 35 °C

Orbitrap MS was operated in the ESI negative mode. Routine calibration was executed weekly to ensure accurate determination (mass accuracy < 5 ppm). Global parameters of ion source were as follows: positive ion spray voltage of 3500 V, negative ion spray voltage of 2000 V, sheath gas flow rate of 35 Arb, aux gas flow rate of 10 Arb, ion transfer tube temperature of 350 °C, vaporizer temperature of 375 °C. MS data were sequentially obtained using full scan (FS) mode and data dependent MS2 (ddMS2) mode. For the FS mode, the mass resolution and scan range were 120,000 and *m/z* 90–500, respectively, whereas for the ddMS2 mode, they were 60,000 and *m/z* 40–500. In the ddMS2 mode, top four ions that are presented in the prior survey scan, FS, are selected for subsequent MS2 analysis. The isolation window for each precursor ion was 0.5 *m/z*. A stepwise collision energy of 20, 40, and 60 eV was applied for fragmentation in the higher-energy C-trap dissociation cell.

LC-MS data was processed using the Thermo Xcalibur software. All analytes were detected and quantified as their deprotonated $[M-H]^-$ molecules. Peak integration and quantification was performed with the FS data that exhibited highest quantitative precision and sensitive feature detection.[23] Relevant MS2 data from the ddMS2 mode was examined for confirmative identification of target analytes. Fragmentation interpretation was assisted by the Sirius software.[24] The linear calibration range in the Orbitrap system was 5–1000 µg/L for all $C_{2–3}$OSs.

S2.3 LC-Qtrap MS analysis parameters and data processing

In the LC-Qtrap MS analysis, the MS/MS transition pair for each analyte consists of the $[M-H]^-$ parent ion and HSO_4^- (*m/z* 97) daughter ion. The optimized compound parameters are listed in Table S3. The optimized source parameters were as follows: negative ion spray voltage of 4000 V, curtain gas flow rate of 30 Arb, high level of collision activated dissociation, flow rates of 40 and 50 Arc for the two ion source gases (i.e., nebulizer and auxiliary gas), and temperature of 550 °C for the auxiliary gas.

LC-MS/MS data acquired by the Qtrap system was processed using the Sciex Analyst Software. The linear range in this analytical system was 5–700 μg/L for HAS and LAS, and 5–500 μg/L for GAS.

Table S3. Optimized MS parameters for MRM transitions using LC-Qtrap-MS.

**Compound**	**Precursor ion**	**Product ion**	**MS/MS transition**	**DP[a] (volts)**	**EP[b] (volts)**	**CE[c] (volts)**	**CXP[d] (volts)**
D17-octyl sulfate (D17-OS)	$C_8D_{17}O_4S^-$	DSO_4^-	226.2/98.0	−31.18	−5.91	−27.91	−11.92
Hydroxyacetone sulfate (HAS)	$C_3H_5O_5S^-$	HSO_4^-	153.1/97.0	−23.47	−14.15	−21.98	−14.94
Glycolic acid sulfate (GAS)	$C_2H_3O_6S^-$	HSO_4^-	155.1/97.0	−28.82	−9.36	−20.07	−25.94
Lactic acid sulfate (LAS)	$C_3H_5O_6S^-$	HSO_4^-	169.0/97.0	−5.78	−10.12	−19.3	−12.37

[a] DP: Declustering Potential; [b] EP: Entrance Potential; [c] CE: Collision Energy; [d] CXP: Collision Cell Exit Potential.

S2.4 Additional discussion of solvent effect

During the early stage of RPLC, the injection solvent's strength (50:50 MeOH/H_2O) surpassed that of the mobile phase strength (1:99 MeOH/H_2O). The mixing of the strong cosolvent (MeOH) from the sample into the water-rich mobile phase at the column head temporarily disturbed the analyte distribution between the stationary phase and mobile phase.[25,26] This disturbance was caused by the swift movement of the MeOH solvent band, which acted as a disruptive force, accelerating the migration of certain molecules.[27] This alteration in elution behavior resulted in peak spreading and bifurcation of HAS and LAS, where the front part of the chromatographic peak resided in the strong solvent strength zone for a longer duration than the rear part. In contrast, GAS, which lacks methyl groups and is the most hydrophilic among the three species, eluted faster or at a similar pace to the solvent front, remaining unaffected by these solvent dynamics. The influence of solvent effect was further confirmed by Figure S4, which shows improved peak shapes of HAS and LAS with a weaker solvent (100% H_2O) while deteriorated peak shapes with a stronger solvent (100% MeOH).

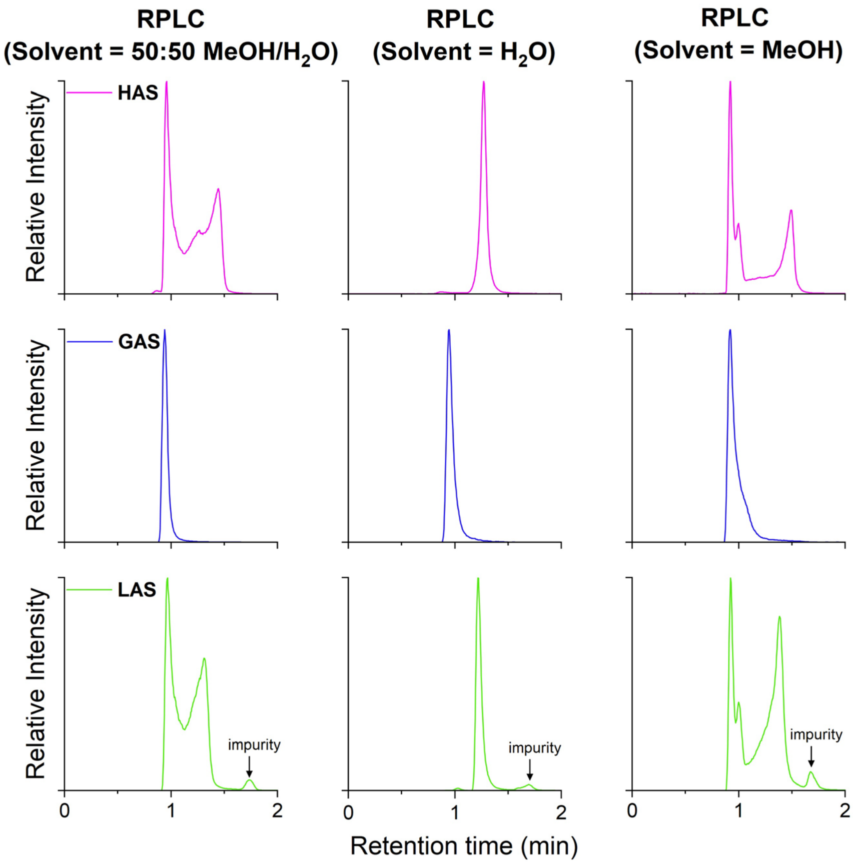

Figure S4. Comparisons of RPLC EICs of HAS, GAS and LAS standards prepared in three different solvents, including 50:50 MeOH/H2O (left column), 100% H_2O (middle column), and 100% MeOH (right column).

S2.5 MS/MS spectra

Figure S5 shows the MS/MS spectra of the four C_{2-3}OSs detected in the ambient samples (i.e., HAS, GAS, LAS and its isomer LAS_i). Plausible fragmentation pathways were proposed based on the interpretation of the product ion generation. At collision energy of 20-40-60 eV, the three carboxylic OSs exhibited a propensity for producing the bisulfate anion (HSO_4^-), which became the predominant peak in the MS2 spectrum. Previous studies that applied different collision energies have also shared such observation.[7,28] The easy formation of HSO_4^- was speculated to be the result of proton transferring from the carboxylic acid group to the sulfate group, followed by homolytic fission of C-O bond.[29,30] For HAS, the bisulfate ion was the second most abundant daughter ion, in which the hydrogen atom was likely donated by the adjacent methyl group (proton on the γ-carbon).[28]

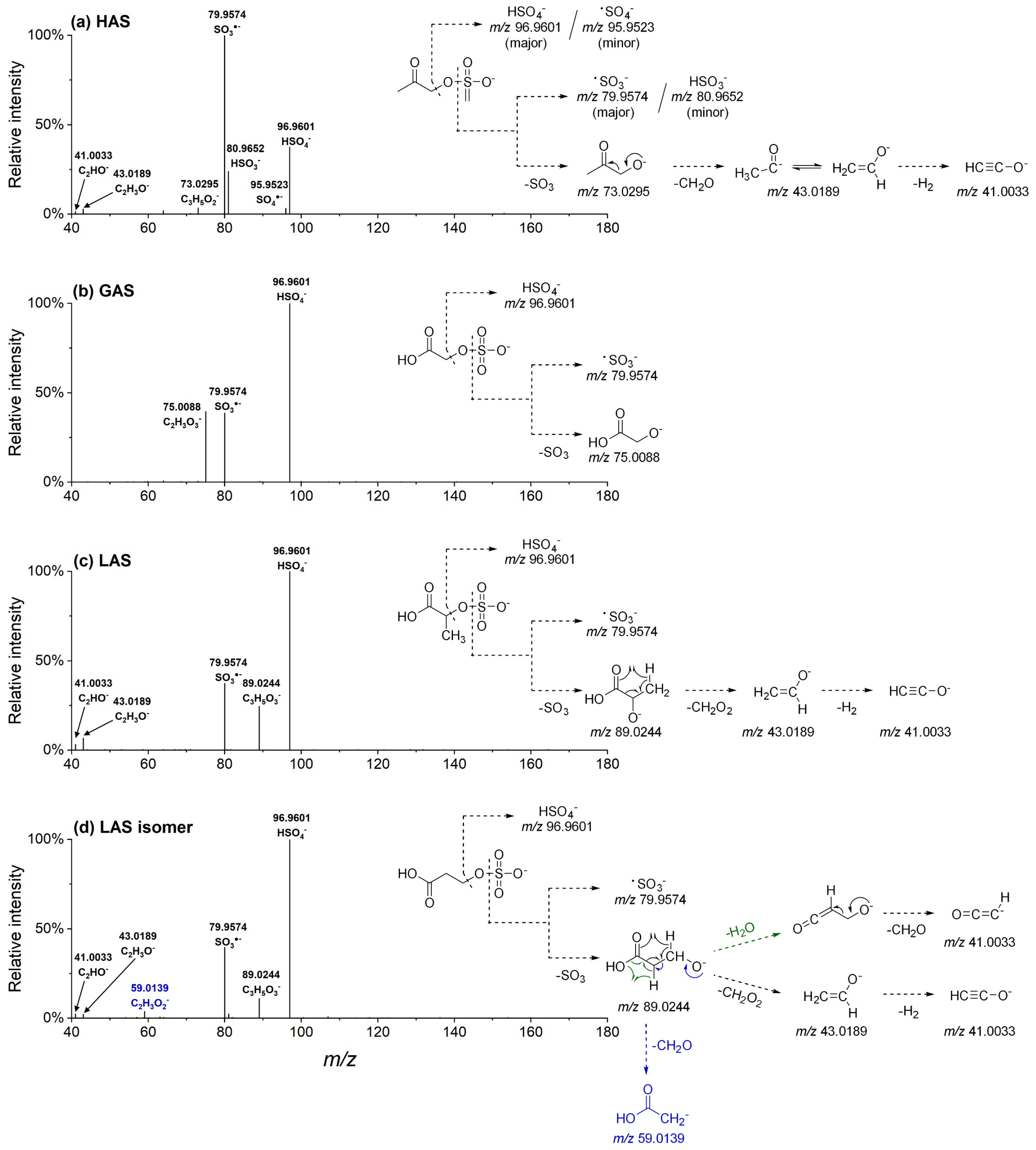

Figure S5. MS/MS spectra and proposed fragmentation pathways of C_{2-3}OSs detected in $PM_{2.5}$ samples.

S3. Supplementary information on internal standards

Figures S6 shows the total ion chromatograms obtained from two columns, illustrating the elution profiles of C_{2-3}OSs and the ISs.

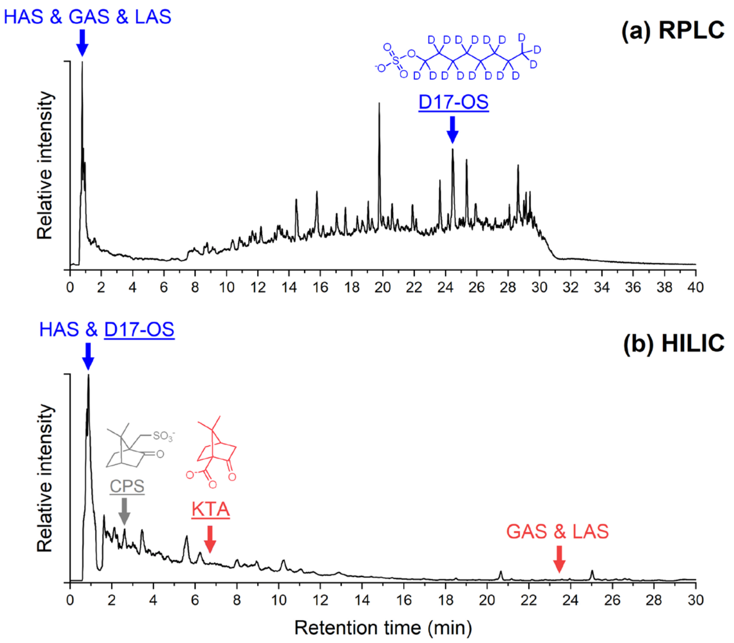

Figure S6. Total ion chromatograms of one demonstration sample obtained from (a) RPLC and (b) HILIC systems. Elution positions of $C_{2\text{–}3}$OSs and ISs (underlined) are indicated with arrows.

The three ISs using the LC-MS analysis, namely D17-OS, CPS, and KTA, are listed in Table S4, along with their formulas, structures, $[M{-}H]^-$ *m/z* values and retention times (RTs) on the two types of columns.

Table S4. Properties of ISs used in this study.

Internal Standard	Formula	Structure	*m/z*	LC system	RT(s) (min)	PA_{max} / PA_{min}[b]	Objects of application
D17-octyl sulfate (D17-OS)	$C_8D_{17}O_4S^-$	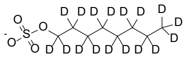	226.1920	RPLC	24.42	3	HAS, GAS, LAS
				HILIC	0.80	65	HAS
Camphorsulfonic acid [a] (CPS)	$C_{10}H_{15}O_4S^-$	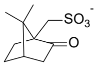	231.0697	HILIC	2.43	270	\
Ketopinic acid (KTA)	$C_{10}H_{13}O_3^-$	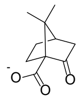	181.0870	HILIC	6.31	13	GAS, LAS

[a] Not applicable after evaluation.
[b] The ratio of the largest PA to the smallest PA across all samples.

While the same amount of ISs were introduced in each sample, their peak areas (PA) showed a variation pattern of higher PA in less polluted samples and lower PA in more polluted samples. This sample-dependency is a clear indication of matrix effects. Next, we used the ratio of the largest PA to the smallest PA (i.e., PA_{max}/PA_{min}) to roughly indicate the fluctuation range of MS response across all samples. D17-OS demonstrated a more variable sample-to-sample response in the HILIC analysis compared to the RPLC analysis, as reflected in the PA_{max}/PA_{min} indicator (65 vs 3, see Table S4). This is expected considering D17-OS eluted from the HILIC column in the solvent zone (Figure S6). KTA provided a relatively more stable

signal across samples than the other two ISs, attributed to its later elution position on the HILIC column. Somehow, CPS exhibited irregular LC-MS behaviors in some samples, presenting distorted peak shapes, significantly attenuated signals, and severely deviating RT (see example EICs in Figure S7). Note that in cases of anomalies, the identification of CPS peak was confirmed by MS2. Using the following criteria as anomaly discriminators—1) RT deviation > 0.5 min, 2) irregular peak shape, and 3) substantially reduced PA—we flagged 14 of the 84 samples as exceptional cases concerning CPS detection. We hypothesize that this atypical behavior may originate from interactions between CPS and concentrated matrix components in samples with relatively higher PM matrix loadings. The underlying mechanism requires further investigation. Consequently, CPS was deemed unsuitable as an IS for HILIC-MS analysis.

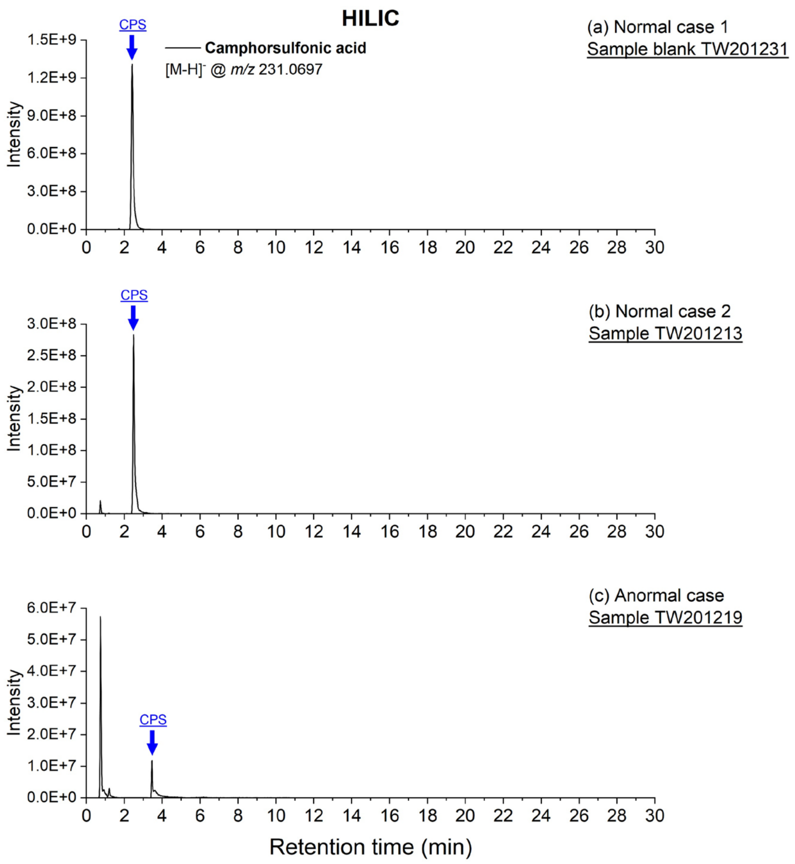

Figure S7. Comparison of EICs of deprotonated CPS obtained with HILIC-Orbitrap MS under different sample conditions.

S4. Comparative analyses of different calibration methodologies

Three types of calibration methods are used, including external standard (ES) calibration, internal standard (IS) calibration, and standard addition (SA) approach. The experimental workflow of the SA approach involved: (1) Preparing triplicates of extraction solution with equal quantity, adding incremental amounts of known analytes to each aliquot and bringing them to the same final volume. Original concentrations were estimated based on the HILIC results, with the total addition controlled to increase the

analytical signal by a factor of around 1.5 to 5; (2) Measuring the LC-MS signal for each solution; (3) Plotting the MS response against the concentration of added standard and extrapolating the linear curve back to the negative x-axis to obtain the actual concentration in the sample. Figure S8 shows the example calibration curves by the three approaches. The IS calibrations in the RPLC-MS method are shown in Panels (a1-a3). Both IS and ES calibrations were made in the HILIC-MS method, shown in Panels (b1-b3) and (c1-c3). In the ES calibration in HILIC-MS analysis, segmented quadratic curves were employed to improve measurement accuracy. SA calibrations, shown in Panels (d1-d3), are performed in the RPLC-MS method in demonstration of the significant impact of matrix effects on quantification via the RPLC-MS method.

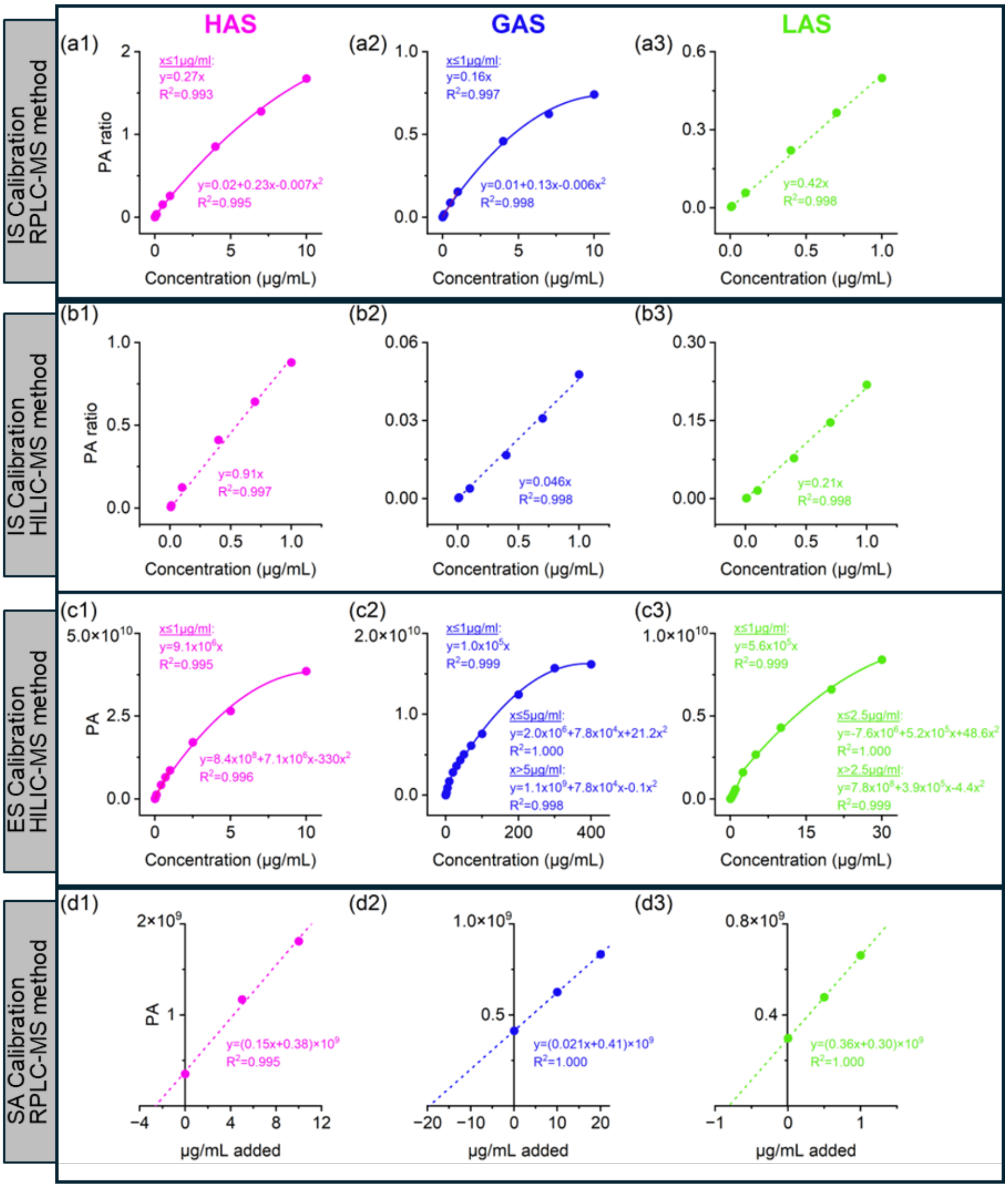

Figure S8. Example calibration curves for three C_{2-3}OSs using linear (dashed line) and quadratic (solid line) regression. Panels (a1-3) show IS calibration in RPLC-MS and panels (b1-3) show IS calibration in HILIC-MS analysis. Panels (c1-3) show ES calibration in HILIC-MS analysis. Panels (d1-3) show SA in RPLC-MS analysis.

Figure S9 compares the calibration curves derived from different matrices in RPLC-MS analysis. Standard solutions of three C_{2-3}OSs yielded higher slopes compared to the sample extracts, with greater signal reduction in more concentrated matrices.

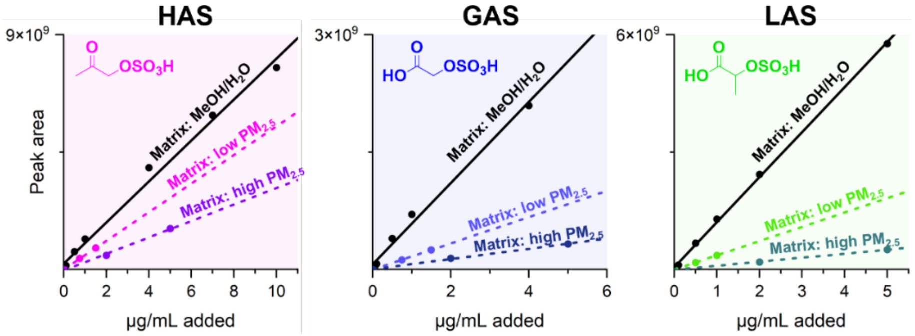

Figure S9. Comparison of the calibration curves for C_{2-3}OSs in standard solution (1:1 MeOH/H_2O) and sample matrices in the RPLC-MS method. To facilitate comparison, all PA have been offset by the lowest PA in each curve

For RPLC-MS analysis, IS calibration was applied to quantify the three C_{2-3}OSs in all samples. In HILIC-MS analysis, we adopted IS calibration for HAS, and ES calibration for GAS and LAS quantification after we have compared the results derived from IS and ES calibrations. The comparison details are described below.

Figure S10 shows the comparative data using one high concentration sample as an illustrative example. HAS, GAS, and LAS were quantified through 5 approaches, all employing HILIC-MS analysis. The five approaches are (1) D17-OS/KTA-based IS calibration curves applied to the diluted extract, (2) ES calibration curves applied to the diluted extract, (3) D17-OS/KTA-based IS calibration curves applied to the concentrated extract (obtained following the original sample processing protocol), with D17-OS as an IS for HAS and KTA as an IS for GAS and LAS, (4) ES calibration curves applied to the concentrated extract, and (5) SA method. For simplicity, we use $C_{D\text{-}IS}$ and $C_{C\text{-}IS}$ to denote concentrations derived using IS calibration on the diluted extract and the concentrated extract, respectively. Other ensuing notations are similarly defined.

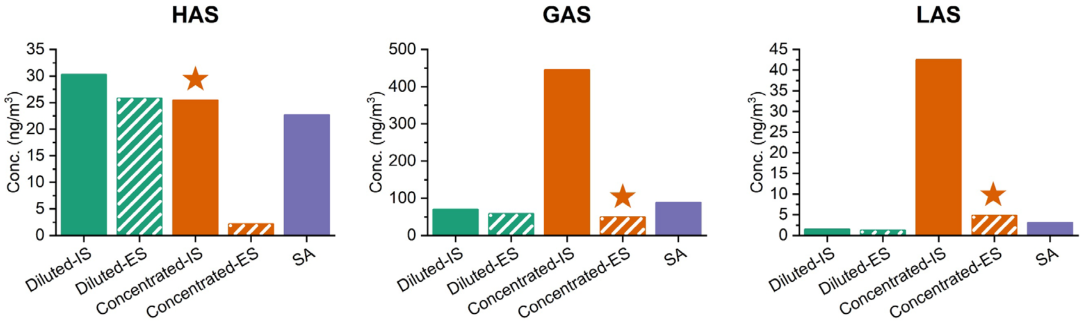

Figure S10. Comparison of OS concentrations generated with different calibration approaches for one demonstration sample. Asterisk-marked approach was used for sample calculations in the HILIC-MS analysis.

Of the five approaches, SA method has taken care of the matrix effects, thus providing the most accurate quantification results. For diluted replicates, IS and ES methods yielded comparable results to those by the SA method. In the case of the concentrated extracts produced from the original processing, $HAS_{C\text{-}IS}$ demonstrated a comparable level with $HAS_{D\text{-}IS}$ and HAS_{SA}. This was likely the result of the co-eluting pair, HAS and D17-OS, being suppressed by the matrix to a similar extent. Using ES calibration, $HAS_{C\text{-}ES}$ on the other hand was seriously underestimated, highlighting the importance of using IS with a similar elution time to efficiently compensate matrix effects arising from the gradient-front region. On the other hand, KTA proved to be an ill-suited IS for GAS and LAS, as KTA eluted in an earlier region with still an appreciable amount of matrix while GAS and LAS eluted in a sufficiently later region on the HILIC column with minimal matrix interference (Figure S6b). As a result, $GAS_{C\text{-}IS}$ and $LAS_{C\text{-}IS}$ were significantly over-estimated (Figure S10). Instead, the ES-based method provided relatively accurate concentrations, evidenced by the comparable concentration levels to those by the SA method.

Hence, we correlated $HAS_{C\text{-}IS}$, $GAS_{C\text{-}ES}$ and $LAS_{C\text{-}ES}$ with their respective concentrations obtained using D17-OS/KTA-based calibration on the dilute replicates (i.e., $OS_{D\text{-}IS}$) for ten samples. The regression slopes and Pearson correlation R values are HAS: slope 0.84, R 0.96; GAS: slope 0.59, R 0.98; and LAS: slope 2.5, R 0.98. The regression slopes were then used to normalize the OS concentrations for all ambient $PM_{2.5}$ samples.

Figure S11 displays scatter plots evaluating the agreement between concentration measurements from the SA reference method (x-axis) and the two LC methods (y-axis).

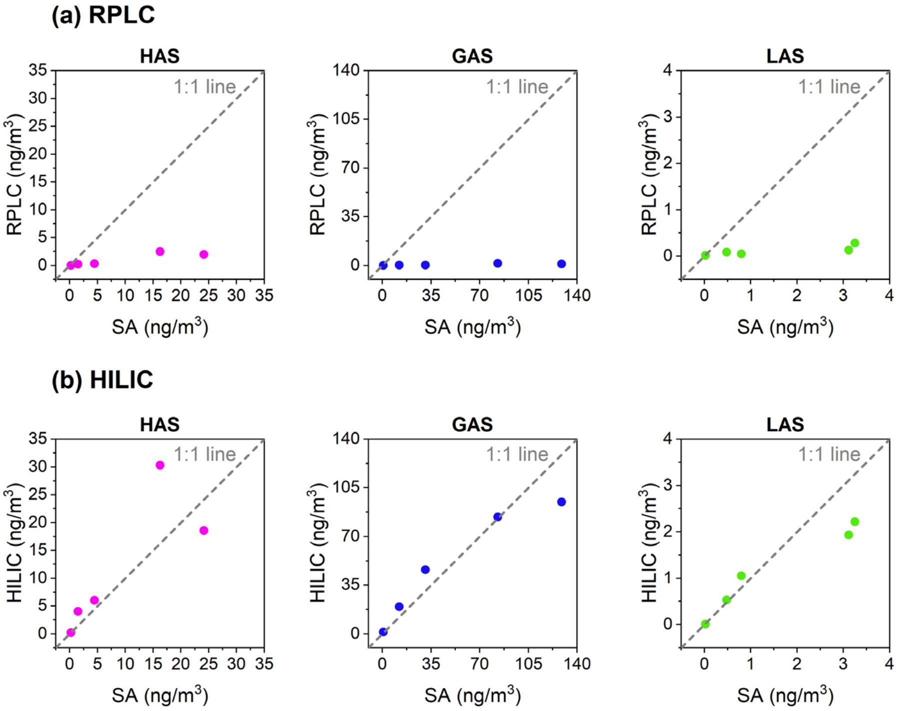

Figure S11. Scatter plots of concentrations determined by (a) RPLC and (b) HILIC methods vs those determined by the SA method. The dotted line represents the 1:1 line of perfect agreement.

S5. Assessing matrix effects in environmental samples

Figure S12 demonstrates that RPLC-derived concentrations exhibited increasing negative bias as $PM_{2.5}$ and sulfate concentrations rose.

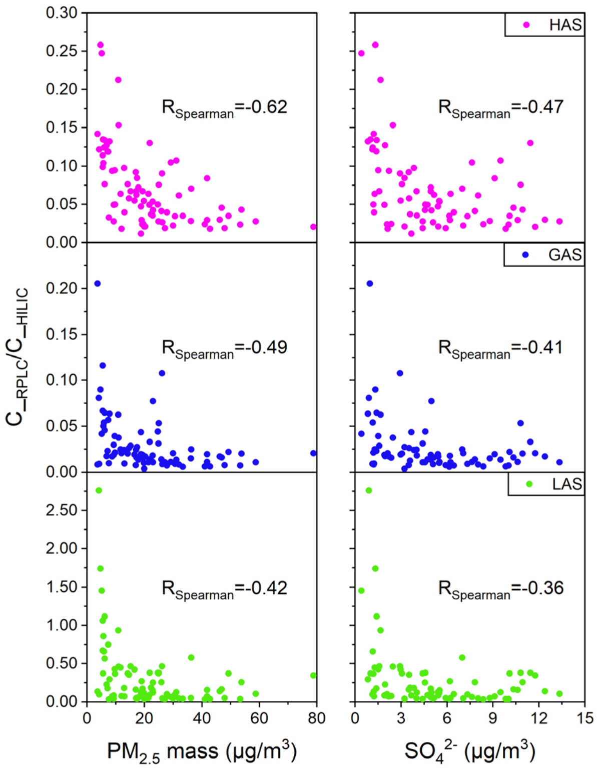

Figure S12. Scatter plots of C_{RPLC}/C_{HILIC} ratio vs $PM_{2.5}$ mass (left panel) and sulfate ion (right panel).

To provide a semi-quantitative assessment of matrix effects (ME), we calculated ME% using the following equation:

$$ME\% = (1 - \frac{C_{x\ in\ sample\ by\ calibration}}{C_{x\ in\ sample\ by\ standard\ addition}}) \times 100$$

where signal abundance is indicated by concentration (C_x) and the analyte is denoted as x. As shown in Figure S13, when the sample loading shifted from low to high, the ME% values of all analytes increased, albeit to varying extents. Among the three $C_{2\text{–}3}$OSs, GAS was the most susceptible to and severely affected by ion suppression, with ME% values exceeding 90% in all samples, including the least polluted one. In contrast, LAS exhibited a more variable ME profile dependent on matrix complexity, with ME% values ranging from 96% in heavily polluted samples to 8% in lightly polluted samples. Dilution of sample extracts, as observed in the two diluted assays presented in Figure S13a, was found to reduce ion suppression. This effect was particularly pronounced for HAS and LAS, resulting in a decrease in ME% values from approximately 90% to less than 60%. For GAS, however, only a minor improvement (~10% decrease of ME%) was observed after dilution, which again underscores its vulnerability to ME.

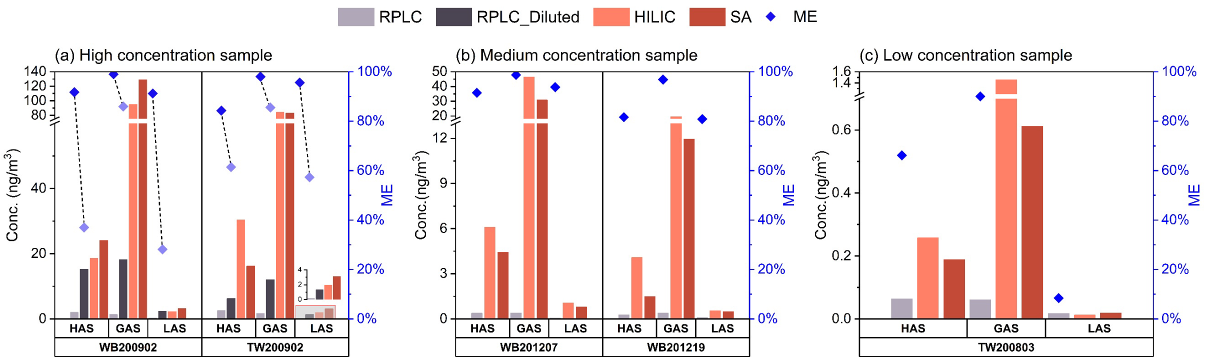

Figure S13. Comparison of results obtained by different methods for C_{2-3}OSs in (a) high concentration samples, (b) medium concentration samples, and (c) low concentration sample.

Figure S14 shows scatter plots of OS concentrations in all ambient samples determined by HILIC-Orbitrap MS method versus those by the RPLC-Orbitrap MS method. The relatively greater divergence in values observed for LAS could be attributed to its greater sensitivity to sample-specific matrix effects, as evidenced by a higher HILIC/RPLC ratio observed at the Tsuen Wan site.

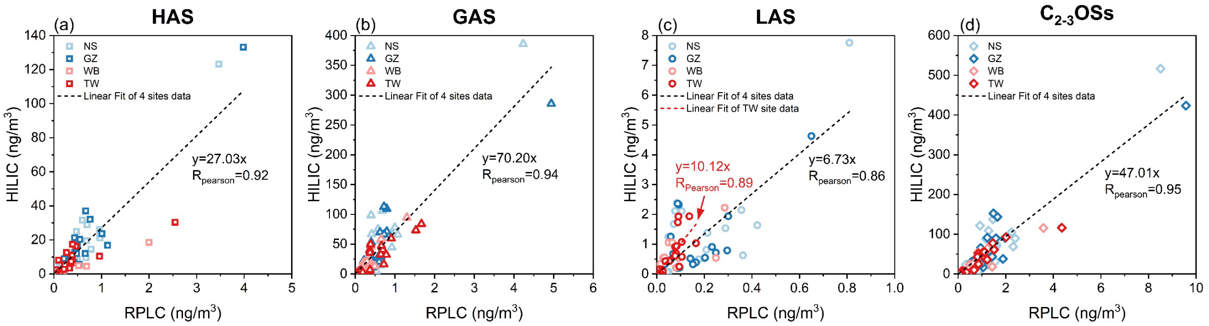

Figure S14. Correlations between HILIC and RPLC measurements for (a) HAS, (b) GAS, (c) LAS and (d) total C_{2-3}OSs. NS: Nansha; GZ: urban Guangzhou; WB: Clear Water Bay; TW: Tsuen Wan.

S6. Investigation of the underestimated measurement bias

S6.1 Investigation of the impact of EDTA on LC-MS response

EDTA, a chelating agent, was added to methanol extract solvent to improve the extraction efficiency of nitroaromatic compounds (another analyte group of interest within our research scope). We conducted comparative experiments to determine whether the inclusion of EDTA during sample extraction inadvertently introduced an exogenous source of ion suppression. Figure S15 presents a schematic sketch of the experimental procedure.

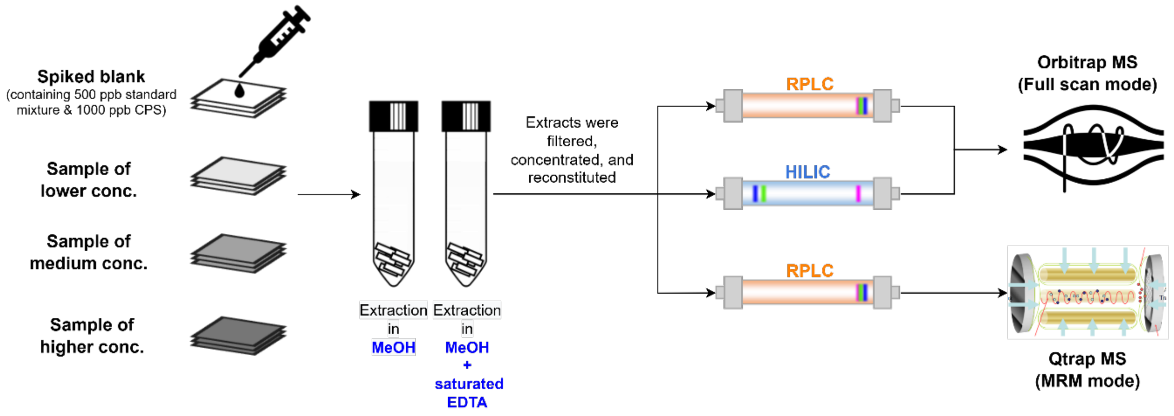

Figure S15. Simplified flow chart of the supplementary experiments.

Three filter samples with varying concentration levels were chosen to obtain the best possible understanding. A fortified blank filter was prepared as a control group to represent a stripped-sample-matrix case. Equal-sized sample/spiked blank filters (30 cm^2 each) were immersed in pure methanol and methanol with saturated EDTA, respectively, making a total of eight aliquots extracted in parallel. After undergoing the subsequent sample pretreatment steps, each reconstituted sample extract was submitted to three types of analysis: RPLC-Orbitrap MS in FS mode, HILIC-Orbitrap MS in FS mode, and RPLC-Qtrap MS in MRM mode.

Figure S16 compares the recoveries of individual $C_{2–3}$OSs spiked on blank filters that were extracted in methanol vs methanol with saturated EDTA and measured by three different LC-MS approaches. Utilizing neat methanol as the extraction solvent, all three LC-MS approaches provided similar and excellent recoveries of the three analytes (~90% or greater). Upon the addition of EDTA, variable outcomes were observed. The HILIC-Orbitrap MS configuration still presented good recovery of all $C_{2–3}$OS species (denoted by filled squares in Figure S16), demonstrating the effectiveness of analyte extraction. With the RPLC column installed, both Orbitrap and Qtrap MS systems displayed signs of ion suppression induced by EDTA to varying extents. Relying on the high-resolution FS data provided by the Orbitrap detector (denoted by filled circles in Figure S16), HAS and LAS were hardly affected, but the GAS signal decreased dramatically, from 95% (without EDTA) to 49% (with EDTA). More staggering suppression was identified in the Qtrap analysis (denoted by hollow circles in Figure S16), wherein the recoveries of HAS, GAS, and LAS all decreased by more than 50%, with GAS showing the largest decrease exceeding 90%.

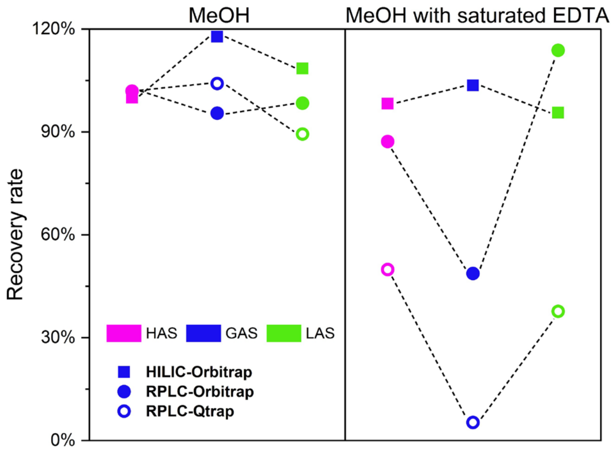

Figure S16. Comparison of recovery of $C_{2–3}OSs$ spiked on blank filters that were extracted using different media (methanol vs methanol saturated with EDTA) and determined by three different combinations of LC and MS configurations.

EDTA, being highly polar due to its tetra-carboxylic acid and di-amine functionalities, showed a retention pattern similar to that of $C_{2–3}OSs$ in the RP columns (Figure S17). This co-elution of chemically ionizable interferents thus invoked a substantial ion suppression effect. While all three species were impacted by EDTA simultaneously, GAS displayed the greatest susceptibility to this interference. This is likely attributed to the unique chemistry of GAS—stronger hydrophilicity and reduced steric hindrance resulting from the absence of a methyl group—that facilitates its interaction with EDTA or other endogenous interferences in the sample. Consequently, GAS was the most vulnerable species in RPLC analysis, potentially explaining its significant underestimation from another aspect.

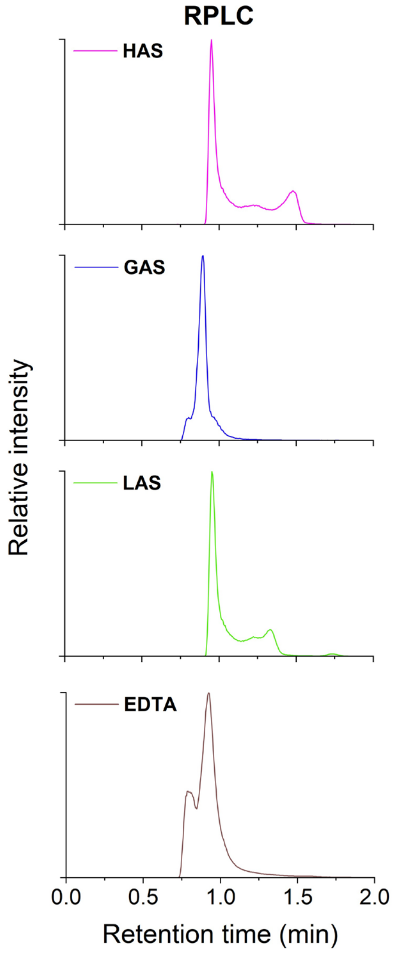

Figure S17. RPLC EICs of deprotonated HAS, GAS, LAS and EDTA obtained from the RPLC-Orbitrap MS system.

For sample detection (case of complex matrix analysis), the presence of EDTA appeared to have a negligible impact on the determined analyte concentrations. This is concluded from Figure S18 showing the scatter plots of HAS, GAS, and LAS measurements in three ambient samples without (w/o) EDTA versus those with (w/) EDTA for each of the three LC-MS approaches. A strong correlation ($R > 0.98$) was observed for all the methods, with linear regression slopes close to one, suggesting that the obtained results were statistically immune to the exogenous suppressor. This disagreement with the spiked blank analysis findings indicates that, under RPLC conditions, the interference caused by EDTA was insignificant when compared to that exerted by the bulk co-extracted $PM_{2.5}$ constituents in real matrices.

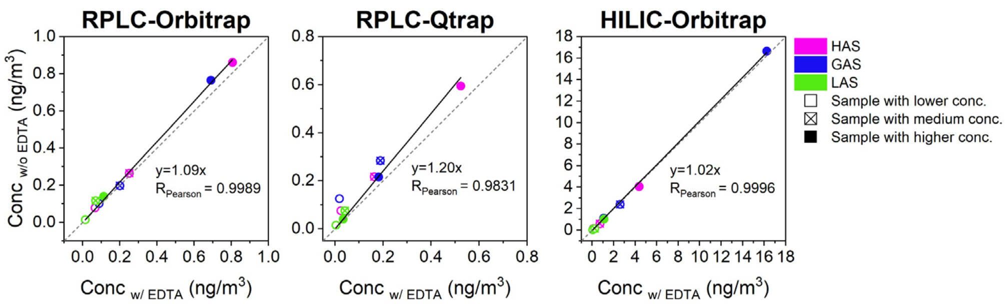

Figure S18. Comparisons of HAS, GAS, and LAS concentrations in three $PM_{2.5}$ samples as determined following sample extraction w/o EDTA vs w/ EDTA in the methanol solvent and measured by three LC-MS configurations. The dotted line represents the 1:1 line of perfect agreement.

S6.2 Investigation of internal factor #1: Bisulfate ion

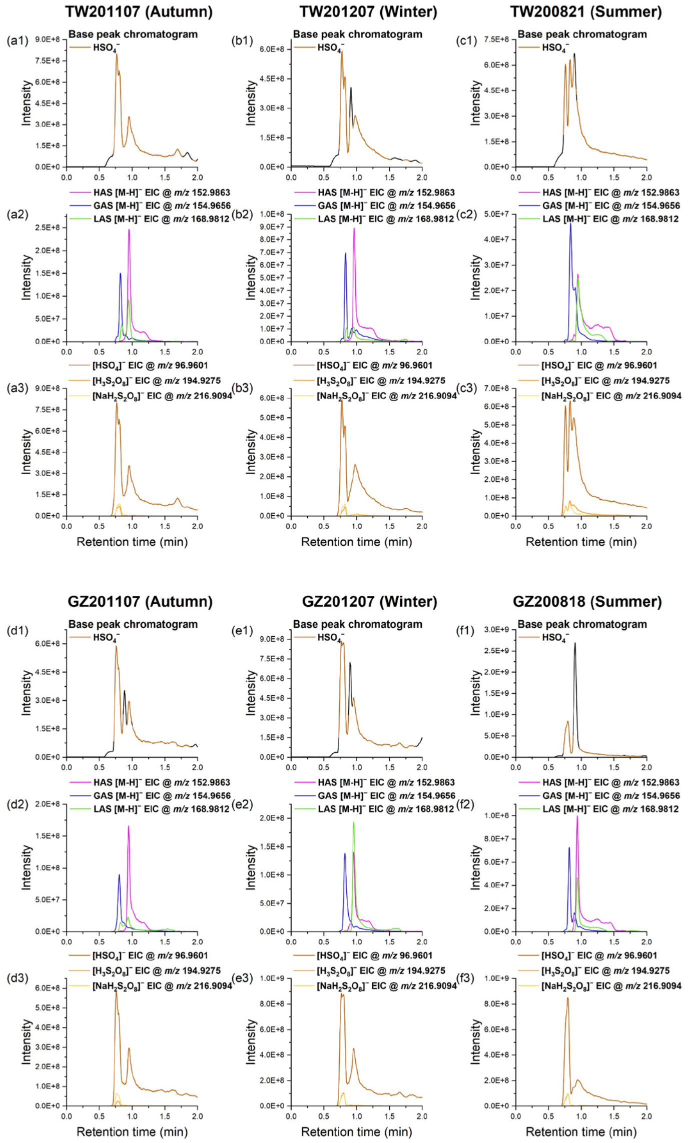

Figure S19. RPLC chromatograms comparing bisulfate ion interference across six field samples collected from different sites (Tsuen Wan, TW; Guangzhou, GZ) and seasons (autumn, winter, summer).

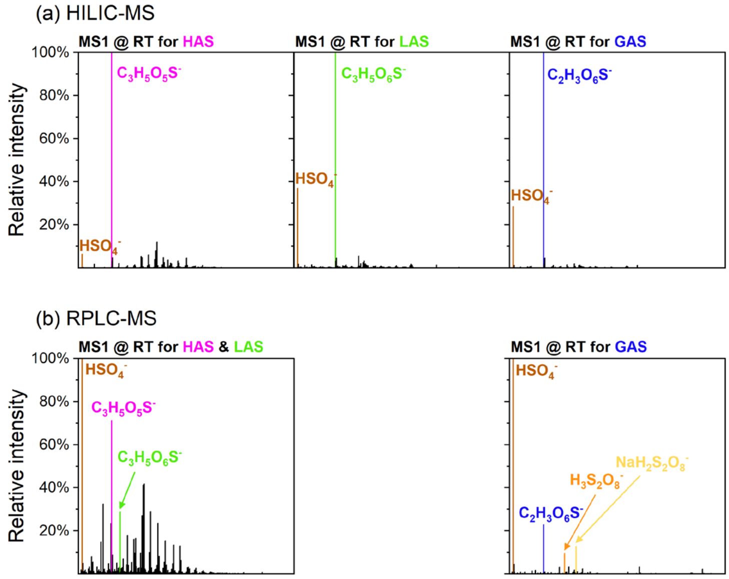

Figure S20. MS1 profiles at the elution RTs of $C_{2–3}$OSs in (a) HILIC-MS and (b) RPLC-MS analyses of a field sample.

S6.3 Investigation of internal factor #2: Adduct formation

We also explored the possibility of ion suppression due to adduct formation with co-existing interfering species. Adduct formation is influenced by various factors, including ionization parameters, analyte structure, and mobile phase composition.[31] The multifunctional structures of the $C_{2–3}$OSs confer on them a propensity to form adducts with certain endogenous substances present in $PM_{2.5}$ samples. For example, GAS and LAS, possessing a carboxylic functional group, can form adducts with humic-like substances through hydrogen bond interactions. With electronegative atoms (e.g., oxygen atoms), $C_{2–3}$OSs readily interact with electropositive component A^{n+} to form corresponding $[M+A]^{n+}$ adducts. Candidates for A^{n+}, such as NH_4^+, Na^+, K^+, are abundant in $PM_{2.5}$. While these positively charged adducts do not directly suppress the $[M–H]^–$ ion signal in the negative mode, they may deplete the concentration of the neutral M molecules in the solution prior to the negative ESI, thereby reducing the formation of the primary deprotonated ion. Furthermore, these electropositive entities can also affect the analyte signal intensity by forming adducts in other anionic forms (e.g., $[M–2H+K]^–$) and altering the overall ionization environment through charge competition and changes in the droplet surface chemistry.

Given the diverse range of possible adduct species, we conducted a focused screening for traces of the most likely adducts. Specifically, we examined associations with sodium, potassium, ammonium, chlorine, bisulfate ion, water, and acetic acid. Among these, a sodium adduct of GAS in the form of $[M–2H+Na]^–$ was successfully observed in the negative ion mode (Figure S21). This adduct likely formed through the direct interaction between the negatively charged sulfate and carboxylic groups with the positively charged sodium ion. Although the detected abundance of this adduct was low, its presence indicates that adduct formation can partially suppress analyte signals. While the observed impact appears minor in this case, it highlights the potential for certain adducts to significantly influence signal distribution, particularly for other analytes not

covered in this study. We therefore recommend that future research further investigate adduct formation and its effects on quantification accuracy in broader analytical contexts.

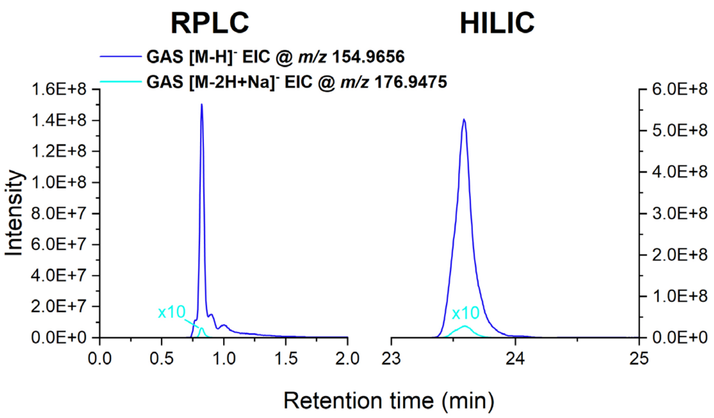

Figure S21. Detection of sodium adduct of GAS [M−2H+Na]⁻ in $PM_{2.5}$ sample.

S6.4 Comparison of ESI-Qtrap and ESI-Orbitrap MS Analyses

The sample analysis described in the preceding section (Section S6.1) also provides an opportunity to compare the two instruments (LC-Qtrap vs LC-Orbitrap MS) in analyzing C_{2-3}OSs. Figure S22 compares the concentrations of HAS, GAS, and LAS determined by RPLC-Qtrap and by RPLC-Orbitrap MS, revealing biased LC-MS/MS measurements compared to the high-resolution FS data when analyzing complex eluents. Such findings indicate that the MRM measurements by Qtrap MS tended to generate lower-quality data for analytes situated in the challenging sections of the chromatogram. Further, a previous LC-ESI-MS/MS study has suggested that MRM can yield false negative results when a molecule can be charged at multiple sites.[32] In our context, it is plausible for both GAS and LAS to undergo deprotonation at two distinct sites—the sulfate group and the carboxylic acid group—at the ESI interface. The resulting isobaric ions (denoted as A and A') would be equally selected and transmitted in the first quadruple, but would fragment differently (i.e., A→B; A'→C). Given that only one transition trace for each analyte (e.g., A→B) is monitored, a lower survival probability for precursor ion A in the sample compared to the standard could potentially result in false negative outcomes.

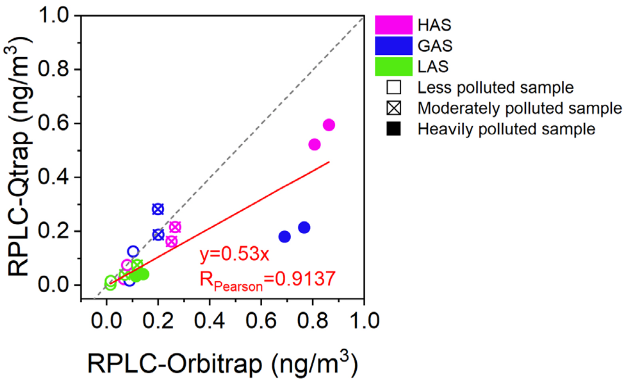

Figure S22. Scatter plot of Qtrap concentration vs Orbitrap concentration.

Figure S23 compares the detection traces of LAS obtained using two MS systems.

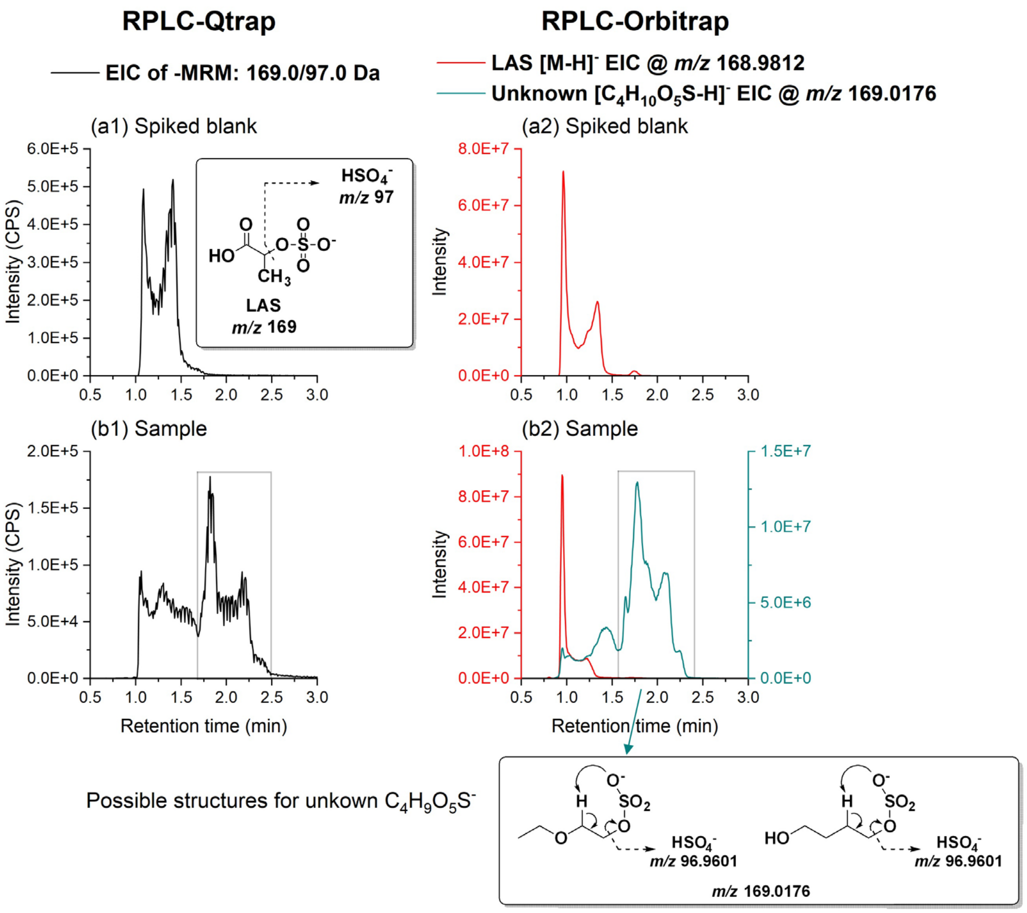

Figure S23. LAS detection by (a) RPLC-Qtrap MS and (b) RPLC-Orbitrap MS.

S7. Analysis of C_{2-3}OSs using HILIC-ESI-Orbitrap MS method

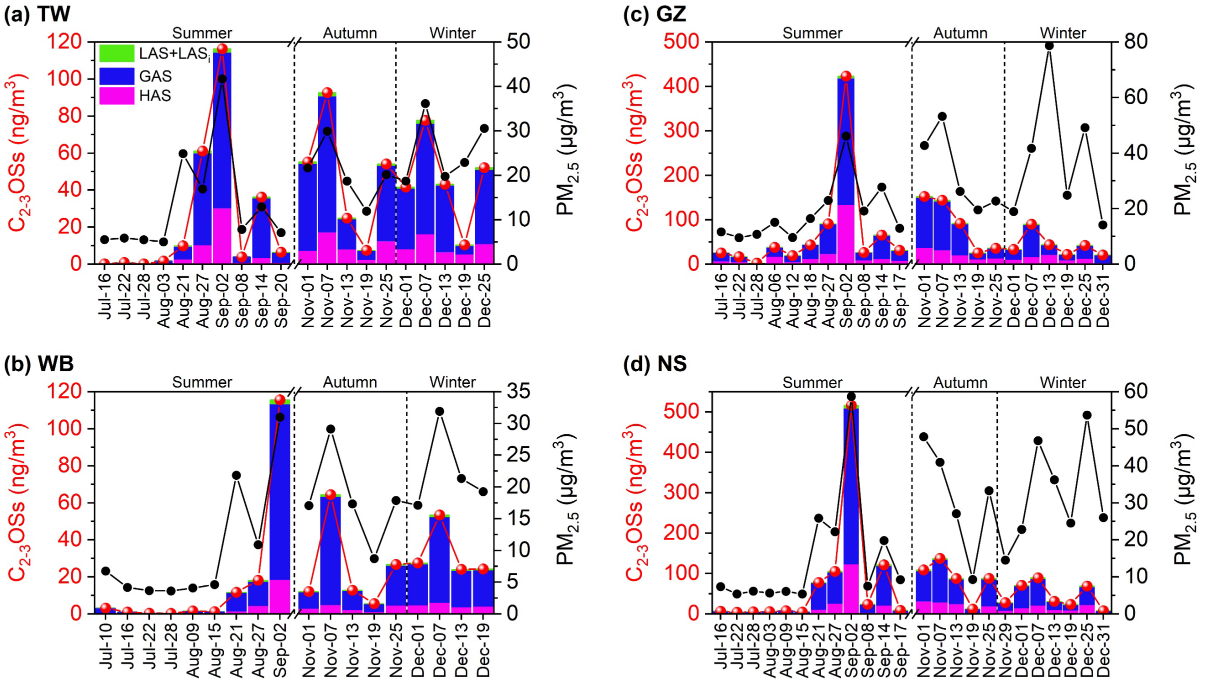

Figure S24. Concentration time series of C_{2-3}OSs and $PM_{2.5}$ at the four sites. TW: Tsuen Wan; WB: Clear Water Bay; GZ: urban Guangzhou; NS: Nansha.

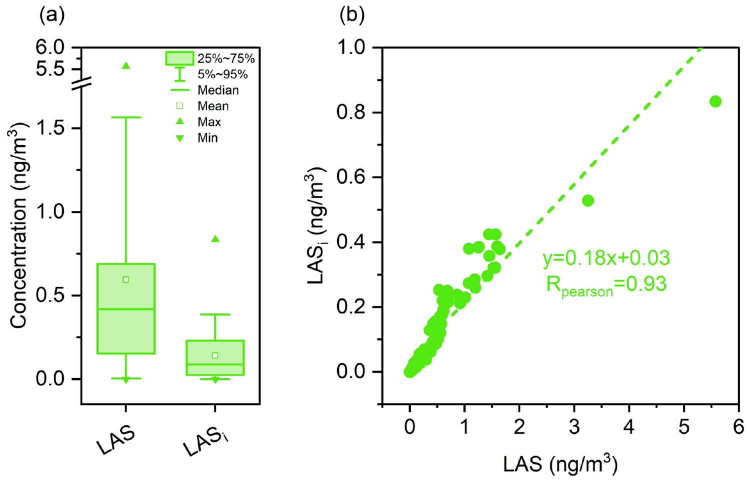

Figure S25. Quantification of LAS and LAS_i using HILIC-MS method: (a) Overall range of concentration data; (b) Correlation of LAS and LAS_i concentrations.

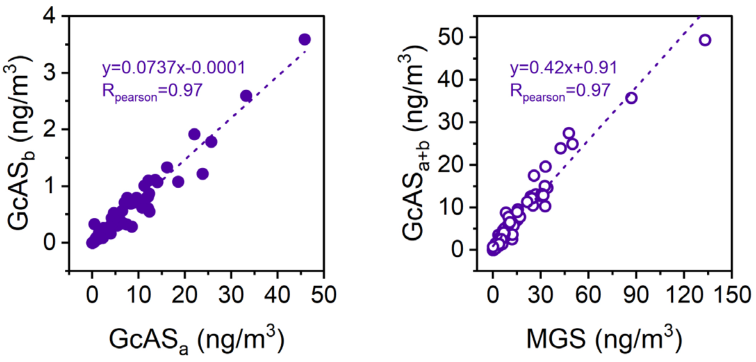

Figure S26. Scatter plots of two GcAS isomers (left) and $GcAS_{a+b}$ vs MGS (right).

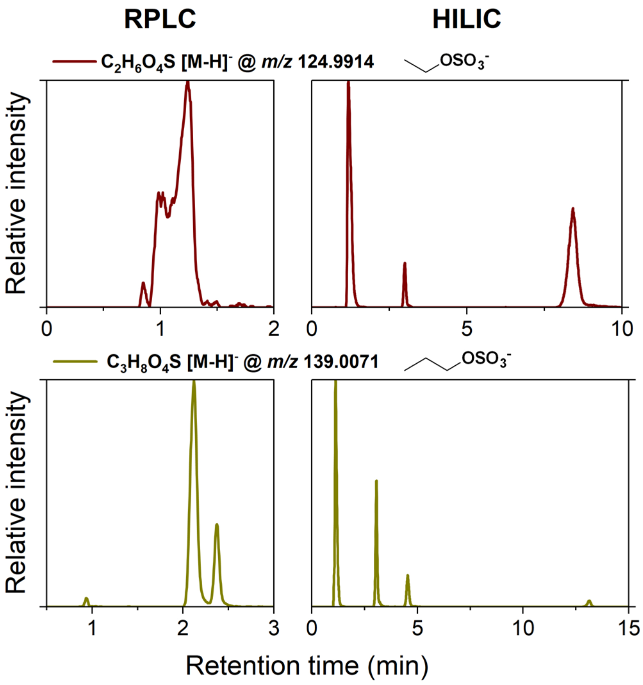

Figure S27. EICs of m/z values corresponding to the deprotonated ethyl sulfate ($C_2H_6O_4S$) (Top row) and propyl sulfate ($C_3H_8O_4S$) (bottom row) obtained using RPLC-MS and HILIC-MS methods. EICs showed no significant peaks outside the displayed RT ranges.

References

(1) Olson, C. N.; Galloway, M. M.; Yu, G.; Hedman, C. J.; Lockett, M. R.; Yoon, T.; Stone, E. A.; Smith, L. M.; Keutsch, F. N. Hydroxycarboxylic Acid-Derived Organosulfates: Synthesis, Stability, and Quantification in Ambient Aerosol. *Environ. Sci. Technol.* **2011**, *45* (15), 6468–6474.

(2) Nguyen, Q. T.; Christensen, M. K.; Cozzi, F.; Zare, A.; Hansen, A. M. K.; Kristensen, K.; Tulinius, T. E.; Madsen, H. H.; Christensen, J. H.; Brandt, J.; Massling, A.; Nøjgaard, J. K.; Glasius, M. Understanding the Anthropogenic Influence on Formation of Biogenic Secondary Organic Aerosols in Denmark via Analysis of Organosulfates and Related Oxidation Products. *Atmospheric Chem. Phys.* **2014**, *14* (17), 8961–8981.

(3) Meade, L. E.; Riva, M.; Blomberg, M. Z.; Brock, A. K.; Qualters, E. M.; Siejack, R. A.; Ramakrishnan, K.; Surratt, J. D.; Kautzman, K. E. Seasonal Variations of Fine Particulate Organosulfates Derived from Biogenic and Anthropogenic Hydrocarbons in the Mid-Atlantic United States. *Atmos. Environ.* **2016**, *145*, 405–414.

(4) Rattanavaraha, W.; Chu, K.; Budisulistiorini, S. H.; Riva, M.; Lin, Y.-H.; Edgerton, E. S.; Baumann, K.; Shaw, S. L.; Guo, H.; King, L.; Weber, R. J.; Neff, M. E.; Stone, E. A.; Offenberg, J. H.; Zhang, Z.; Gold, A.; Surratt, J. D. Assessing the Impact of Anthropogenic Pollution on Isoprene-Derived Secondary Organic Aerosol Formation in $PM_{2.5}$ Collected from the Birmingham, Alabama, Ground Site during the 2013 Southern Oxidant and Aerosol Study. *Atmospheric Chem. Phys.* **2016**, *16* (8), 4897–4914.

(5) Martinsson, J.; Monteil, G.; Sporre, M. K.; Kaldal Hansen, A. M.; Kristensson, A.; Eriksson Stenström, K.; Swietlicki, E.; Glasius, M. Exploring Sources of Biogenic Secondary Organic Aerosol Compounds Using Chemical Analysis and the FLEXPART Model. *Atmospheric Chem. Phys.* **2017**, *17* (18), 11025–11040.

(6) Hettiyadura, A. P. S.; Jayarathne, T.; Baumann, K.; Goldstein, A. H.; de Gouw, J. A.; Koss, A.; Keutsch, F. N.; Skog, K.; Stone, E. A. Qualitative and Quantitative Analysis of Atmospheric Organosulfates in Centreville, Alabama. *Atmospheric Chem. Phys.* **2017**, *17* (2), 1343–1359.

(7) Huang, R.-J.; Cao, J.; Chen, Y.; Yang, L.; Shen, J.; You, Q.; Wang, K.; Lin, C.; Xu, W.; Gao, B.; Li, Y.; Chen, Q.; Hoffmann, T.; O'Dowd, C. D.; Bilde, M.; Glasius, M. Organosulfates in Atmospheric Aerosol: Synthesis and Quantitative Analysis of $PM_{2.5}$ from Xi'an, Northwestern China. *Atmospheric Meas. Tech.* **2018**, *11* (6), 3447–3456.

(8) Wang, Y.; Hu, M.; Guo, S.; Wang, Y.; Zheng, J.; Yang, Y.; Zhu, W.; Tang, R.; Li, X.; Liu, Y.; Le Breton, M.; Du, Z.; Shang, D.; Wu, Y.; Wu, Z.; Song, Y.; Lou, S.; Hallquist, M.; Yu, J. The Secondary Formation of Organosulfates under Interactions between Biogenic Emissions and Anthropogenic Pollutants in Summer in Beijing. *Atmospheric Chem. Phys.* **2018**, *18* (14), 10693–10713.

(9) Glasius, M.; Bering, M. S.; Yee, L. D.; Sá, S. S. de; Isaacman-VanWertz, G.; Wernis, R. A.; Barbosa, H. M. J.; Alexander, M. L.; Palm, B. B.; Hu, W.; Campuzano-Jost, P.; Day, D. A.; Jimenez, J. L.; Shrivastava, M.; Martin, S. T.; Goldstein, A. H. Organosulfates in Aerosols Downwind of an Urban Region in Central Amazon. *Environ. Sci. Process. Impacts* **2018**, *20* (11), 1546–1558.

(10) Hettiyadura, A. P. S.; Al-Naiema, I. M.; Hughes, D. D.; Fang, T.; Stone, E. A. Organosulfates in Atlanta, Georgia: Anthropogenic Influences on Biogenic Secondary Organic Aerosol Formation. *Atmospheric Chem. Phys.* **2019**, *19* (5), 3191–3206.

(11) Hughes, D.; Stone, E. Organosulfates in the Midwestern United States: Abundance, Composition and Stability. *Environ. Chem.* **2019**, *16*, 312–322.

(12) Cai, D.; Wang, X.; Chen, J.; Li, X. Molecular Characterization of Organosulfates in Highly Polluted Atmosphere Using Ultra-High-Resolution Mass Spectrometry. *J. Geophys. Res. Atmospheres* **2020**, *125* (8), e2019JD032253.

(13) Hughes, D. D.; Christiansen, M. B.; Milani, A.; Vermeuel, M. P.; Novak, G. A.; Alwe, H. D.; Dickens, A. F.; Pierce, R. B.; Millet, D. B.; Bertram, T. H.; Stanier, C. O.; Stone, E. A. PM2.5 Chemistry, Organosulfates, and Secondary Organic Aerosol during the 2017 Lake Michigan Ozone Study. *Atmos. Environ.* **2021**, *244*, 117939.

(14) Chen, Y.; Dombek, T.; Hand, J.; Zhang, Z.; Gold, A.; Ault, A. P.; Levine, K. E.; Surratt, J. D. Seasonal Contribution of Isoprene-Derived Organosulfates to Total Water-Soluble Fine Particulate Organic Sulfur in the United States. *ACS Earth Space Chem.* **2021**, *5* (9), 2419–2432.

(15) Glasius, M.; Thomsen, D.; Wang, K.; Iversen, L. S.; Duan, J.; Huang, R.-J. Chemical Characteristics and Sources of Organosulfates, Organosulfonates, and Carboxylic Acids in Aerosols in Urban Xi'an, Northwest China. *Sci. Total Environ.* **2022**, *810*, 151187.

(16) Ding, S.; Chen, Y.; Devineni, S. R.; Pavuluri, C. M.; Li, X.-D. Distribution Characteristics of Organosulfates (OSs) in PM2.5 in Tianjin, Northern China: Quantitative Analysis of Total and Three OS Species. *Sci. Total Environ.* **2022**, *834*, 155314.

(17) Kanellopoulos, P. G.; Kotsaki, S. P.; Chrysochou, E.; Koukoulakis, K.; Zacharopoulos, N.; Philippopoulos, A.; Bakeas, E. PM2.5-Bound Organosulfates in Two Eastern Mediterranean Cities: The Dominance of Isoprene Organosulfates. *Chemosphere* **2022**, *297*, 134103.

(18) Wang, Y.; Ma, Y.; Kuang, B.; Lin, P.; Liang, Y.; Huang, C.; Yu, J. Z. Abundance of Organosulfates Derived from Biogenic Volatile Organic Compounds: Seasonal and Spatial Contrasts at Four Sites in China. *Sci. Total Environ.* **2022**, *806*, 151275.

(19) Wang, Y.; Liang, S.; Le Breton, M.; Wang, Q. Q.; Liu, Q.; Ho, C. H.; Kuang, B. Y.; Wu, C.; Hallquist, M.; Tong, R.; Yu, J. Z. Field Observations of C2 and C3 Organosulfates and Insights into Their Formation Mechanisms at a Suburban Site in Hong Kong. *Sci. Total Environ.* **2023**, *904*, 166851.

(20) Schoenberger, T. Determination of Standard Sample Purity Using the High-Precision 1H-NMR Process. *Anal. Bioanal. Chem.* **2012**, *403* (1), 247–254.
(21) Weber, M.; Hellriegel, C.; Rück, A.; Sauermoser, R.; Wüthrich, J. Using High-Performance Quantitative NMR (HP-qNMR®) for Certifying Traceable and Highly Accurate Purity Values of Organic Reference Materials with Uncertainties <0.1 %. *Accreditation Qual. Assur.* **2013**, *18* (2), 91–98.
(22) Saito, N.; Kitamaki, Y.; Otsuka, S.; Yamanaka, N.; Nishizaki, Y.; Sugimoto, N.; Imura, H.; Ihara, T. Extended Internal Standard Method for Quantitative 1H NMR Assisted by Chromatography (EIC) for Analyte Overlapping Impurity on 1H NMR Spectra. *Talanta* **2018**, *184*, 484–490.
(23) Guo, J.; Huan, T. Comparison of Full-Scan, Data-Dependent, and Data-Independent Acquisition Modes in Liquid Chromatography–Mass Spectrometry Based Untargeted Metabolomics. *Anal. Chem.* **2020**, *92* (12), 8072–8080.
(24) Dührkop, K.; Fleischauer, M.; Ludwig, M.; Aksenov, A. A.; Melnik, A. V.; Meusel, M.; Dorrestein, P. C.; Rousu, J.; Böcker, S. SIRIUS 4: A Rapid Tool for Turning Tandem Mass Spectra into Metabolite Structure Information. *Nat. Methods* **2019**, *16* (4), 299–302.
(25) Layne, J.; Farcas, T.; Rustamov, I.; Ahmed, F. Volume-Load Capacity in Fast-Gradient Liquid Chromatography: Effect of Sample Solvent Composition and Injection Volume on Chromatographic Performance. *J. Chromatogr. A* **2001**, *913* (1), 233–242.
(26) Keunchkarian, S.; Reta, M.; Romero, L.; Castells, C. Effect of Sample Solvent on the Chromatographic Peak Shape of Analytes Eluted under Reversed-Phase Liquid Chromatogaphic Conditions. *J. Chromatogr. A* **2006**, *1119* (1), 20–28.
(27) Loeser, E.; Drumm, P. Using Strong Injection Solvents with 100% Aqueous Mobile Phase in RP-LC. *J. Sep. Sci.* **2006**, *29* (18), 2847–2852.
(28) Hettiyadura, A. P. S.; Stone, E. A.; Kundu, S.; Baker, Z.; Geddes, E.; Richards, K.; Humphry, T. Determination of Atmospheric Organosulfates Using HILIC Chromatography with MS Detection. *Atmospheric Meas. Tech.* **2015**, *8* (6), 2347–2358.
(29) Safi Shalamzari, M.; Ryabtsova, O.; Kahnt, A.; Vermeylen, R.; Hérent, M.-F.; Quetin-Leclercq, J.; Van der Veken, P.; Maenhaut, W.; Claeys, M. Mass Spectrometric Characterization of Organosulfates Related to Secondary Organic Aerosol from Isoprene. *Rapid Commun. Mass Spectrom.* **2013**, *27* (7), 784–794.
(30) Attygalle, A. B.; García-Rubio, S.; Ta, J.; Meinwald, J. Collisionally-Induced Dissociation Mass Spectra of Organic Sulfate Anions. *J. Chem. Soc. Perkin Trans. 2* **2001**, No. 4, 498–506.
(31) Erngren, I.; Haglöf, J.; Engskog, M. K. R.; Nestor, M.; Hedeland, M.; Arvidsson, T.; Pettersson, C. Adduct Formation in Electrospray Ionisation-Mass Spectrometry with Hydrophilic Interaction Liquid Chromatography Is Strongly Affected by the Inorganic Ion Concentration of the Samples. *J. Chromatogr. A* **2019**, *1600*, 174–182.
(32) Kaufmann, A.; Butcher, P.; Maden, K.; Widmer, M.; Giles, K.; Uría, D. Are Liquid Chromatography/Electrospray Tandem Quadrupole Fragmentation Ratios Unequivocal Confirmation Criteria? *Rapid Commun. Mass Spectrom.* **2009**, *23* (7), 985–998.
